# Supplementary material for: Diagnostic accuracy of perinatal post-mortem ultrasound (PMUS): a systematic review
Source: BMJ Paediatr Open. 2019 Nov 11;3(1):e000566. doi: 10.1136/bmjpo-2019-000566 (PMC6863669; doi:10.1136/bmjpo-2019-000566)

Appendix

S1. Search strategy for retrieval of included studies

MEDLINE:

#	01 January 1998 to 31 December 2018	Results (n)
1	(Autopsy [Mesh] OR autopsy [tiab] OR necropsy [tiab] OR post-mortem [tiab] OR postmortem [tiab])	148316
2	(Infant death, sudden [Mesh] OR infant* [tiab] OR child*[tiab] OR adolescent [tiab] OR pediatric*[tiab] OR paediatric*[tiab] OR neonate*[tiab] OR newborn*[tiab] OR baby [tiab] OR babies [tiab] OR youth*[tiab] OR perinatal OR fetal [tiab] OR foetal [tiab] OR termination* [tiab] OR abortion* [tiab])	2276140
3	("Ultrasonography"[Mesh] OR ultraso*[tiab] OR sonograph*[tiab] OR echo*[tiab] OR imaging [tiab] OR radiology [tiab])	1308153
**4**	1 and 2 and 3	**3133**

EMBASE:

#	01 January 1998 to 31 December 2018; Human subjects only	Results (n)
1	Autopsy.a.f	68338
2	(postmortem or post-mortem).a.f	33839
3	1 or 2	87723
4	(child* or preschool child* or adolescent or infant* or newborn* or pediatric* or infant* or newborn* or neonat* or baby or babies or paediatric* or adoles* or teen* or youth* or fetal or foetal).a.f.	2900920
5	(ultraso* or sonograph* or echo*).a.f.	778476
**6**	**3 and 4 and 5**	**3915**

COCHRANE LIBRARY

#	01 January 1998 to 31 December 2018	Results (n)
1	Autopsy OR postmortem OR post-mortem (Word variations have been searched)	1181
2	child OR adolescent OR infant OR newborn OR pediatric OR neonate OR baby OR babies OR paediatric OR teen OR youth OR fetal OR foetal (Word variations have been searched)	240101
3	Ultrasonography OR ultraso* OR sonograph* OR echo* OR imaging OR radiology (Word variations have been searched)	102777
**4**	**#1 and #2 and #3**	**135**

S2. SROC plot of sensitivity against false positivity rate (1 – specificity) for all studies (excluding the publication by Kang et al 2019) for diagnostic accuracy rate for overall diagnosis. Bivariate overall summary estimates of sensitivity and false positivity rate are overlaid with corresponding 95% confidence ellipses

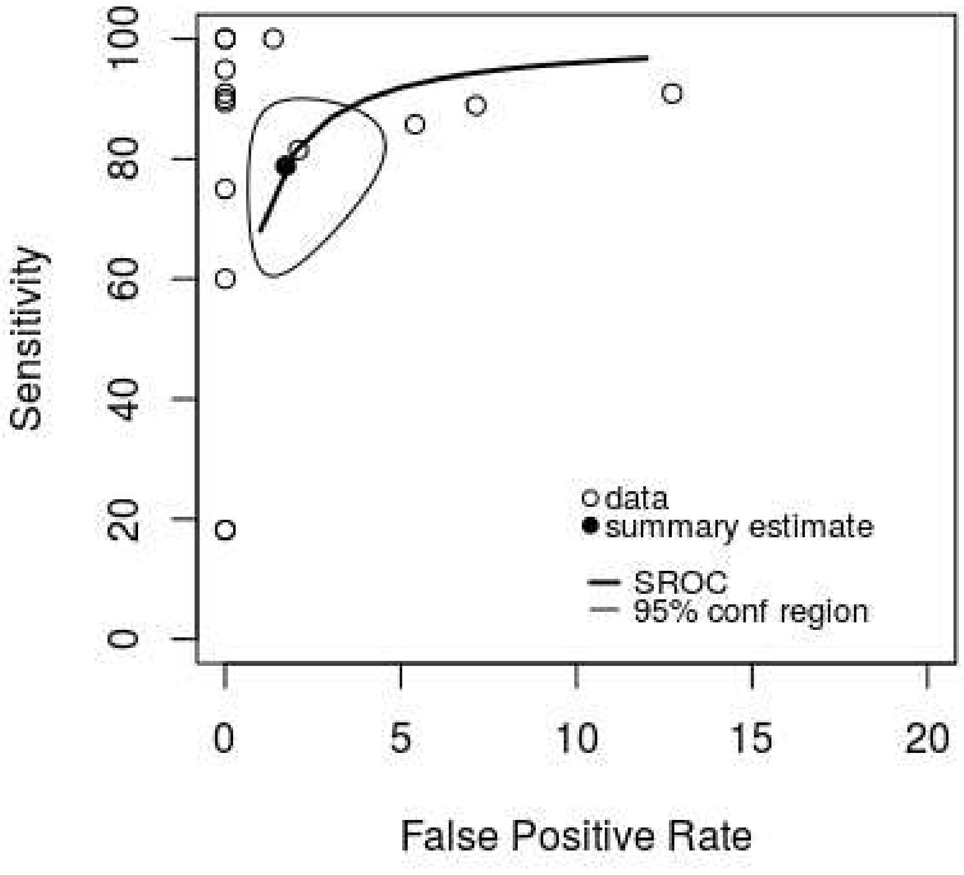

S3. SROC plot of sensitivity against false positivity rate (1 – specificity) for all studies (excluding the publication by Kang et al 2019) for diagnostic accuracy results per body system. Bivariate overall summary estimates of sensitivity and false positivity rate are overlaid with corresponding 95% confidence ellipses.

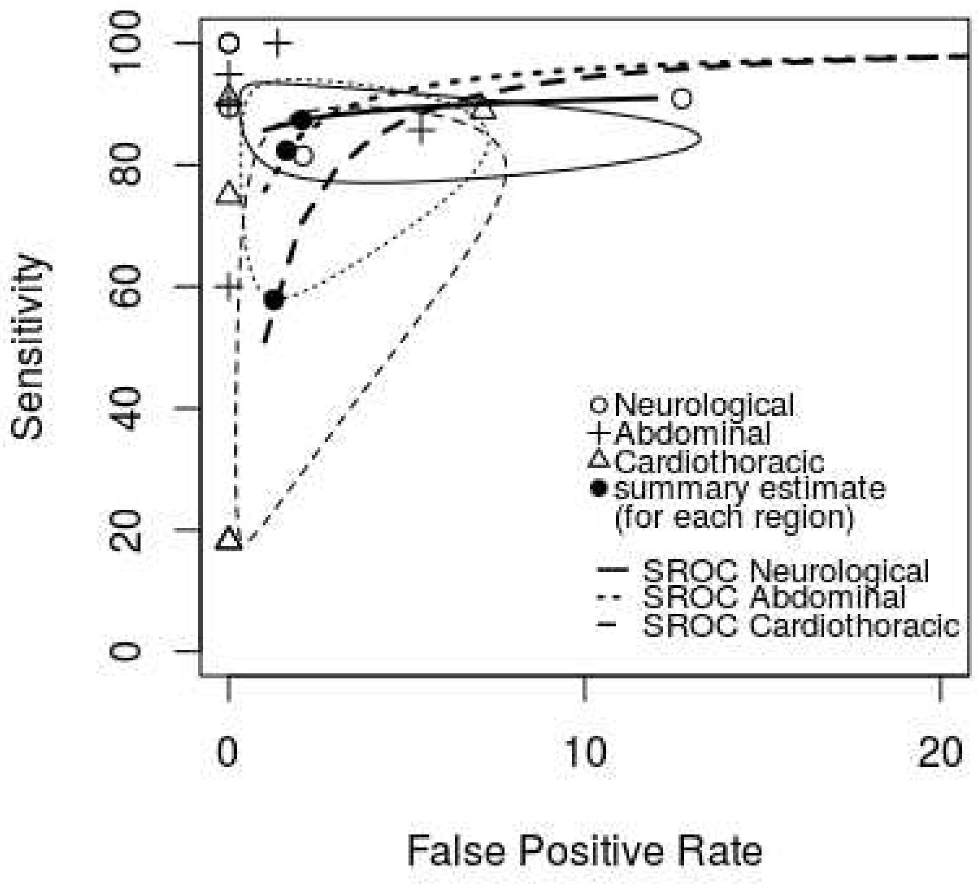

Supplement: Supplementary data [file bmjpo-2019-000566supp001.pdf]
