# Supplementary material for: Diagnostic accuracy of perinatal post-mortem ultrasound (PMUS): a systematic review
Source: BMJ Paediatr Open. 2019 Nov 11;3(1):e000566. doi: 10.1136/bmjpo-2019-000566 (PMC6863669; doi:10.1136/bmjpo-2019-000566)
Supplement: Author's manuscript [file bmjpo-2019-000566.draft_revisions.pdf]

BMJ Paediatrics Open

BMJ Paediatrics Open is committed to open peer review. As part of this commitment we make the peer review history of every article we publish publicly available.

When an article is published we post the peer reviewers' comments and the authors' responses online. We also post the versions of the paper that were used during peer review. These are the versions that the peer review comments apply to.

The versions of the paper that follow are the versions that were submitted during the peer review process. They are not the versions of record or the final published versions. They should not be cited or distributed as the published version of this manuscript.

BMJ Paediatrics Open is an open access journal and the full, final, typeset and author-corrected version of record of the manuscript is available on our site with no access controls, subscription charges or pay-per-view fees (http://bmjpaedsopen.bmj.com).

If you have any questions on BMJ Paediatrics Open's open peer review process please email info.bmjpo@bmj.com

BMJ Paediatrics Open

Diagnostic Accuracy of Paediatric Post-mortem Ultrasound (PMUS): A Systematic Review

Journal:	*BMJ Paediatrics Open*
Manuscript ID	bmjpo-2019-000566
Article Type:	Original article
Date Submitted by the Author:	13-Aug-2019
Complete List of Authors:	Shelmerdine, Susan; Great Ormond Street Hospital for Children, Clinical Radiology; UCL GOSH ICH, Radiology<br>Langan, Dean; UCL GOSH ICH<br>Sebire, Neil; Great Ormond Street Hospital for Children, Paediatric Pathology; UCL GOSH ICH<br>Arthurs, Owen; Great Ormond Street Hospital for Children, Clinical Radiology; UCL GOSH ICH
Keywords:	Imaging, Pathology, General Paediatrics, Fetal Medicine, Evidence Based Medicine

SCHOLARONE™
Manuscripts

Title Page

Full Title:

Diagnostic Accuracy of Paediatric Post-mortem Ultrasound (PMUS): A Systematic Review

Short Title:

Diagnostic Accuracy of Post-mortem Ultrasound

Authors:

Susan Cheng Shelmerdine, MBBS, FRCR [a, b] ; susan.shelmerdine@gosh.nhs.uk

Dean Langan, PhD [b, c] ; d.langan@ucl.ac.uk

Neil James Sebire, Prof, MD [b, d] ; neil.sebire@gosh.nhs.uk

Owen John Arthurs, PhD [a, b] ; owen.arthurs@gosh.nhs.uk

Affiliations:

a Department of Clinical Radiology, Great Ormond Street Hospital for Children, London, UK

b UCL Great Ormond Street Institute of Child Health, Great Ormond Street Hospital for Children, London, UK.

c Centre of Applied Statistics Courses, University College London, London, UK

d Department of Paediatric Pathology, Great Ormond Street Hospital for Children, London, UK

Corresponding Author:

Name: Dr. Susan Shelmerdine

Job title: Paediatric Radiology Research Fellow

Address: Department of Clinical Radiology,

Great Ormond Street Hospital for Children, London, United Kingdom

Email: susan.shelmerdine@gosh.nhs.uk

Telephone: +44 (0) 20 9200 (x6369)

Fax: Not applicable.

Key words

Autopsy, minimally invasive autopsy, paediatric, perinatal, ultrasound

Confidential: For Review Only

What is known about the subject:

- Whole body post-mortem ultrasound is being performed in several European centres for perinatal deaths.
- Post-mortem ultrasound can be a useful alternative technique for non-invasive autopsy, where MRI is unavailable.

What this study adds:

- Ultrasound provides a high diagnostic accuracy for neurological and abdominal pathologies.
- Assessment of cardiothoracic anomalies is challenging and less accurate.

Confidential: For Review Only

ABSTRACT:

Abstract:

Objective:

Ultrasound is ubiquitous in live paediatric imaging, however its usage in post-mortem setting is less established. This systematic review aims to evaluate the diagnostic accuracy of paediatric post-mortem ultrasound, PMUS.

Design:

MEDLINE, Embase and Cochrane Library databases were queried for studies published between 1998 – 2018 assessing PMUS diagnostic accuracy rates in children <18 years old, using autopsy as reference standard. Risk of bias was assessed using QUADAS 2. A random-effects model was used to obtain pooled estimates of sensitivity, specificity for different body systems.

Results:

Four studies were included, all relating to ultrasound for perinatal deaths. The pooled diagnostic sensitivity and specificity for neurological abnormalities was 85% (95% CI: 72%, 93%; $I^2 = 59\%$) and 97% (86%, 99%; $I^2 = 79\%$) ; for cardiothoracic abnormalities 52% (26%, 78%, $I^2 = 79\%$) and 97% (87%, 99%; $I^2 = 81\%$); and for abdominal abnormalities 80% (61%, 91%; $I^2 = 63\%$) and 98% (90%, 99%; $I^2 = 87\%$) . Combining all body systems, the pooled sensitivity and specificity was 74% (60%, 85%; $I^2 = 80\%$) and 97% (93%, 99%; $I^2 = 81\%$).

Conclusions:

PMUS demonstrates a reasonable diagnostic accuracy, particularly for abdominal and neurological abnormalities, although cardiac anomalies were less readily identified.

Trial registration:

This study was registered in PROSPERO International prospective register of systematic reviews, CRD42018106968.

KEYWORDS:

Autopsy, minimally invasive autopsy, paediatric, perinatal, ultrasound

Confidential: For Review Only

ABBREVIATIONS:

PRISMA Preferred Reported Items for Systematic Reviews and Meta-Analysis statement

PROSPERO International Prospective Register of Systematic Reviews

PMUS Post-mortem ultrasound

PMMR Post-mortem magnetic resonance imaging

PMI Post-mortem interval (i.e. time between death and imaging)

INTRODUCTION:

Post-mortem imaging techniques in children have gained popularity over the past decade, particularly given the decline in parental consent rates for invasive autopsy [1, 2]. Factors for this are multifaceted but include religious, emotional and communication barriers combined with the continued desire for further information to aid in future pregnancy management and bereavement counselling [3].

The majority of studies relating to post-mortem imaging in children have related to post-mortem MRI (PMMR), with a recently published systematic review [4] reporting sensitivity rates of 0.73 (95% CI 0.56 – 0.84) in a pooled sample of 953 children across eight studies. For post-mortem CT imaging (PMCT), there have been fewer studies in children compared to adults, with the majority reporting CT to be of lower yield in identification for the cause of death in non-suspicious (i.e. 'natural' deaths) and perinatal losses [5-7], with a slight increase in benefit within a forensic setting [8, 9].

Ultrasound, whilst ubiquitous in live paediatric imaging, is far less established in the post-mortem setting. There are only a few articles either describing single site experience [10], or focussing on single anatomical areas such as congenital cardiac disorders [11]. With rising costs, radiographer staffing shortages and reduced availability and access to MRI and CT imaging, ultrasound may play a key role in a future non-invasive post-mortem imaging service. The equipment is cheaper to acquire and run than cross-sectional imaging, with a variety of healthcare specialists already skilled in sonographic techniques (e.g. fetal medicine clinicians, obstetricians, radiologists, sonographers). Nevertheless, wi3thout robust evidence detailing the diagnostic accuracy of this technique, it remains difficult to counsel parents appropriately and for clinicians to understand the benefit of this modality as a non-invasive imaging alternative to conventional autopsy. The aim of this systematic review is therefore to

https://mc.manuscriptcentral.com/bmjpo

Confidential: For Review Only

comprehensively assess the diagnostic accuracy of paediatric post-mortem ultrasonography (PMUS) from the existing literature.

MATERIALS AND METHODS:

This study was registered in PROSPERO International prospective register of systematic reviews, CRD42018106968[12]. PRISMA (Preferred Reporting Items for Systematic reviews and Meta-Analyses) guidelines for transparent reporting of systematic reviews were followed [13]. Ethical approval was not required for this retrospective review of published data.

Patient and Public Involvement

Patients were not directly involved in the design of this systematic review, although prior work by our research group[3] using patient representatives and focus group meetings revealed significant support for the development and further research into non-invasive methods of childhood autopsy.

Literature Review

We searched MEDLINE (Ovid), EMBASE and the Cochrane Library databases for eligible articles published between 1 January 1998 and 31 December 2018 (20 year range), using database specific Boolean search strategies with terms and word variations relating to 'autopsy', 'ultrasonography' and 'paediatrics'. The full search strategy was conducted in December 2018 (See Appendix S1 for details). A repeat search was conducted in July 2019 did not reveal any further eligible manuscripts for inclusion.

Eligibility Criteria

Inclusion criteria encompassed work investigating diagnostic accuracy of post-mortem ultrasound imaging using autopsy as a reference standard. Studies were limited to human subjects, including fetuses (any gestation) and children (ages 0- 18 years). No restrictions were placed on method of ultrasound technique, machine vendor, and experience of operator or type of clinical setting. No language restrictions were used.

Exclusion criteria included studies reported as conference abstracts, case reports, editorials, opinion articles, pictorial reviews, multimedia files (online videos, podcasts) and small case series (fewer than 5 cases). Articles

Confidential: For Review Only

without autopsy reference standard, or those relating to diagnostic accuracy for antenatal ultrasound imaging were excluded.

All articles were independently searched by two reviewers (both paediatric radiologists with >8 years (SCS) and >12 years (OJA) experience). Abstracts of suitable studies were examined, and full papers were obtained. References from the retrieved full text articles were manually examined for other possible publications. Disagreements were resolved by consensus.

Data extraction and Quantitative Data Synthesis

Two reviewers (SCS, OJA) independently extracted data from the full articles into a database (Excel, Microsoft, Redmond WA, USA) which included the following factors: study design, study setting, population demographics, sample size, index test, study methodology, use of reference standard, diagnostic accuracy outcomes, measure of bias.

Raw numbers of true and false positives and negative post-mortem ultrasound diagnoses were collected and inputted into a 2 x 2 table, considering autopsy as reference standard. Missing data were calculated if possible and, unpublished data or further information was obtained by contacting the authors.

Methodological Quality

The quality and measure of bias for each included study was assessed by the modified Quality Assessment of Diagnostic Accuracy Studies (QUADAS 2) criteria [14]. Disagreements were resolved by consensus review of the literature.

Statistical Analysis

Statistical analysis was performed by an experienced statistician (DL) using R [15]. The available data was used to compute sensitivity, specificity, positive predictive value (PPV) and negative predictive value (NPV) for each study. A random-effects model (using REML to estimate the heterogeneity variance) was used to obtain pooled estimates of sensitivity and specificity with 95% confidence intervals for overall PMUS diagnosis and, by body systems (neurological, cardiothoracic, abdominal). The function metaprop within the R package meta was used to synthesise the evidence [16]. Heterogeneity between studies was assessed using the I^2 statistic.

https://mc.manuscriptcentral.com/bmjpo

Confidential: For Review Only

Confidential: For Review Only

RESULTS:

Eligible Studies

The initial search performed on 1 January 2019 yielded 6017 articles, after removal of duplicate studies. On the basis of study title and abstract, 5999 articles were excluded and 18 articles were retrieved in full text. After review of the full text, four studies were eventually included in the systematic review [17-20]. A PRISMA flowchart is shown in **Figure 1**.

Patient Population

Our patient and study characteristics are provided in **Tables 1 and 2**. All four articles related to post-mortem ultrasound for perinatal deaths [17-20]. In all studies, parental consent was given for the ultrasound imaging. In total, 495 fetuses were imaged (sample size range: 75 - 169) and of these, 455 underwent an autopsy as a reference standard. The gestational ages ranged from 11 – 48 weeks. Most of the fetuses were the result of terminations of pregnancy (363/495, 73%), with 11% (52/495) miscarriages and 16% (80/495) intrauterine deaths. Gender mix was not reported in any of the studies.

Confidential: For Review Only

Table 1: Demographic details of population studied for all articles

Author, year	Country	Sample Size, n	Patient Group	Median gestation at death	Gestational age range (weeks)	Median post-mortem weight (g)	Weight range (g)	Mode of Death
**Prodhomme, 2015**	France	169	Fetuses	27 weeks	15 – 38	Not stated	Not stated	97% (164) terminations<br>3% (5) intrauterine deaths
**Kang, 2017**	Belgium & U.K.	163 imaged, 123 with reference test	Fetuses	23 weeks	13 – 42	Not stated	Not stated	50% (82) terminations<br>21% (34) miscarriages<br>29% (47) intrauterine deaths
**Tuchtan, 2018**	France	75	Fetuses	Not stated, however 42 cases were <24 weeks; 33 were >24 weeks	15 – 38	Not stated	Not stated	79% (59) terminations<br>4% (3) miscarriages<br>17% (13) intrauterine deaths
**Votino, 2018**	Belgium	88	Fetuses	21 weeks	11 – 40	702	7 – 4020	66% (58) terminations<br>17% (15) miscarriages<br>17% (15) intrauterine deaths

Confidential: For Review Only

Table 2: Study characteristics for articles included in systematic review

Author, year	Country	Sample Size	Study Design	Number of Centres	Patient Selection	Study Period	Index Test	Reference Test
**Prodhomme, 2015**	France	169	Retrospective	Single	Unclear	4 years (2009 – 13)	2D whole body ultrasound	Conventional autopsy
**Kang, 2017**	Belgium & U.K.	163	Prospective	Multiple, 2 centres	Consecutive	2 years (2014-16)	2D whole body ultrasound	Conventional and minimally invasive autopsy
**Tuchtan, 2018**	France	75	Prospective	Single	Consecutive	1 year (2014)	2D whole body ultrasound	Conventional autopsy
**Votino, 2018**	Belgium	88	Prospective	Single	Consecutive	19 months (2012 – 13)	2D and 3D whole body ultrasound	Conventional autopsy

Confidential: For Review Only

The majority of studies (3/4, 75%) were prospective in design and conducted in a single centre (3/4, 75%). Two studies originated from France [18, 19], one study originated from Belgium [20] and one was a multicentre study involving patients from two institutions, one in the United Kingdom and one in Belgium [17]. The timeframe for data collection ranged between 1 – 4years.

Imaging Assessment and Operator

Details regarding the index (post-mortem ultrasound) and reference (autopsy) tests in the studies of this systematic review are summarised in **Tables 3 and 4**.

Confidential: For Review Only

Table 3: Details of index test for studies included for systematic review.

Author, year	Ultrasound Machine	Ultrasound Transducers/ Probes	Imaging time (mins)	Patient preparation	Ultrasound Operator, Experience	No. operators	Blinded to clinical history	Time between delivery to imaging	Diagnostic accuracy subgroup measures
**Prodhomme, 2015**	Phlips iU22	5-8 MHz microconvex<br>5-12 MHz linear<br>5-17 MHz linear	Not stated	No additional preparation over cold storage of body	Paediatric radiologist, >10 years' experience	Single	No	Not stated	Agreement with final autopsy diagnosis. List of diagnoses given.
**Kang, 2017**	GE Voluson E8; GE LOGIQ E9; Samsung HM70A	GE Voluson:<br>6-18MHz linear<br>6-12MHz curved<br>5-9MHz curved<br><br>GE Logiq:<br>2.5 – 8MHz linear<br>1 – 5MHz curved<br><br>Samsung:<br>7-16 MHz linear	Not stated	No additional preparation over cold storage of body	Fetal medicine doctor or paediatric radiologist (>5years experience each)	Two (one from each centre)	Yes	Median time 2 days (0 – 39 days)	19 internal organs assessed and grouped into 4 anatomic regions for analysis: neurological (brain/spine), thorax, heart and abdomen.<br><br>Overall and individual body system accuracy rates given.
**Tuchtan, 2018**	Toshiba Aplio 500; Supersonic Aixplorer; GE Voluson E8	Toshiba:<br>7-10MHz curved<br>10-15MHz linear<br><br>Supersonic:<br>6-10MHz curved<br>11-15MHz linear<br><br>GE:<br>5-10MHz curved<br>10-14MHz linear	20	No additional preparation over cold storage of body	Paediatric Radiologist, 15years ultrasound and 8 years post-mortem imaging experience	Single	Yes	Not stated	Anatomical structures were divided into seven categories: brain, spine, lung, heart, skeletal, gastrointestinal, and genitourinary.<br><br>Overall and individual body organ accuracy rates given.
**Votino, 2018**	GE Voluson E8	6-18MHz linear,<br>6-12MHz curved,<br>5-9MHz curved	15	Fetuses either fixed in formalin (>15 weeks gestation), otherwise no	Fetal medicine doctor, 10 years of ultrasound experience	Singe	No	Median time 2 days (1 hour - 4 days)	Diagnoses categorised into three body systems: neurological (brain/spine), thorax

Confidential: For Review Only

				additional preparation.					(including heart) and abdomen.<br><br>Individual body system accuracy rates given.

Confidential: For Review Only

Table 4: Details of the reference test for studies included for systematic review.

Author, year	Reference Test	Autopsy Standards	Person(s) performing reference test, experience	Blinded to antenatal clinical history	Blinded to index (ultrasound) test results	Median time between imaging or delivery and autopsy
**Prodhomme, 2015**	Conventional autopsy	Not stated	Pathologists, experience not quantified	No	Not stated	Not stated
**Kang, 2017**	Conventional autopsy or minimally invasive autopsy	Conventional autopsy guidelines (SOFFoeT and Royal College of Pathologists)<br>Or<br>Minimally invasive endoscopic methods described by Sebire N et al, 2012	Paediatric pathologists, >15 years' experience	No	Yes	5 days from delivery to autopsy (0 – 47 days)
**Tuchtan, 2018**	Conventional autopsy	French National Authority of Health guidelines	Pathologists, experience not quantified	No	Yes	Not stated
**Votino, 2018**	Conventional autopsy	Societé française de foetopathologie (SOFFoeT, France) guidelines	Pathologists with 20 years' experience	No	Yes	1 day from US to autopsy (1 hour – 2 days)

Confidential: For Review Only

A variety of ultrasound machine vendors and models were used. The most popular ultrasound machine was Voluson E8 (GE Medical Systems, Zipf, Austria), used by 3/5 (60%) centres. High frequency linear probes were used by all operators, with 3/5 (60%) centres also using curvilinear or micro-convex ultrasound probes to acquire two-dimensional (2D) images. One study also acquired three dimensional (3D) ultrasound volumes for head and body imaging in addition to standard 2D views [20].

A single operator performed all post-mortem ultrasound studies in each centre, and for all cases the ultrasound was performed prior to autopsy (therefore without knowledge of the reference test results). In two studies (2/4, 50%) the operators were blinded to the antenatal history [17, 19], and in the remainder the antenatal results were known to the operator [18, 20]. The operator was frequently a paediatric radiologist (3/5, 60%) or fetal medicine specialist with ultrasound experience (2/5, 40%).

Reference Test

For all studies the reference standard was conventional autopsy. Only one study described a subset of patients (11/123, 9%) who underwent a minimally invasive autopsy by endoscopic examination [17], using a technique described by Sebire et al [21]. Three of the studies reported that their autopsies were performed according to national guidelines (i.e. Royal College of Pathologists [22, 23], SOFFoeT [24], French National Authority of Health [25]). In one study the autopsy guideline was not mentioned [18].

All autopsies were performed by one of several pathologists within a department, rather than a single individual. The pathologists were aware of the clinical and antenatal history for all cases, and in three of the four studies the pathologists were blinded to the ultrasound results.

Methodological Quality Assessment

Using the QUADAS-2 tool, there was a moderate to high risk of bias concerning the 'index text' domain because the persons performing the ultrasound examination in two studies were aware of the patient's antenatal history, and therefore could have been biased when performing the examination [18, 20]. The risk of bias regarding the 'flow and timing' domain were unclear for two other studies since timing between ultrasound examination and autopsy were not defined [18, 19].

https://mc.manuscriptcentral.com/bmjpo

Confidential: For Review Only

In one study [18], the risk of bias was unclear for both the patient selection and also reference standard, given that it was not stated how patients were recruited into the study or whether the autopsy results could have been biased by the ultrasound findings. Assessment of bias regarding overall applicability concerns was low **(Figure 2).**

Diagnostic Accuracy of ultrasound by body system

The diagnostic accuracy rates for sensitivity and specificity of each body system are summarised in **Table 5**, with the results given in forest plots in **Figures 3 and 4** for sensitivity and specificity respectively.

Confidential: For Review Only

Table 5: Estimated sensitivities and specificities of different body parts/ systems from studies included in systematic review, with 95% confidence intervals (CI) in square brackets. ND – non-diagnostic, TP – true positive, TN – true negative, FP - false positive, FN – false negative, NA – not applicable

Author, year	Body Part	Total sample size	Sample size with autopsy	Excluded			TP	FN	FP	TN	Sensitivity, % [95% CI]	Specificity, % [95% CI]
				ND autopsy	ND PMUS	No autopsy						
**Neurological**												
**Votino, 2018**	Brain	88	66	17	4	5	10	1	7	48	90.9 [58.7, 99.8]	87.3 [75.5, 94.7]
**Prodhomme, 2015**	Brain	169	169	0	0	NA	84	10	0	75	89.4 [81.3, 94.8]	100 [95.2, 100]
**Tuchtan, 2018**	Brain	75	75	0	0	NA	22	5	1	47	81.5 [63.3, 91.8]	97.9 [89.1, 99.6]
**Prodhomme, 2015**	Spine	169	169	0	0	NA	18	0	0	151	100 [81.4, 100]	100 [97.5, 100]
**Tuchtan, 2018**	Spine	75	75	0	0	NA	10	0	0	65	100 [72.2, 100]	100 [94.4, 100]
**Kang, 2017**	Brain & Spine	123	70	53	13	NA	13	8	11	38	61.9 [38.4, 81.9]	77.6 [63.4, 88.2]
**Cardiothoracic**												
**Prodhomme, 2015**	Thoracic	169	169	0	0	NA	10	1	0	158	90.9 [58.7, 99.8]	100 [97.7, 100]
**Kang, 2017**	Thoracic	123	123	0	21	NA	5	14	20	84	26.3 [9.2, 51.2]	80.8 [71.9, 87.8]
**Tuchtan, 2018**	Thoracic	75	75	0	0	NA	6	2	0	67	75.0 [40.9, 92.9]	100 [94.6, 100]
**Prodhomme, 2015**	Cardiac	169	169	0	0	NA	2	9	0	158	18.2 [2.3, 51.8]	100 [97.6, 100]
**Kang, 2017**	Cardiac	123	122	1	24	NA	13	13	18	78	50.0 [29.9, 70.1]	73.6 [64.1, 81.7]
**Tuchtan, 2018**	Cardiac	75	75	0	0	NA	4	18	0	53	18.2 [7.3, 38.5]	100 [93.2, 100]
**Votino, 2018**	Cardiac & Thoracic	88	88	0	2	NA	16	2	5	65	88.9 [65.3, 98.6]	92.8 [84.1, 97.6]

Confidential: For Review Only

**Abdominal**												
**Votino, 2018**	Whole Abdomen	88	88	0	2	NA	12	2	4	70	85.7 [57.2, 98.2]	94.6 [86.7, 98.5]
**Kang, 2017**	Whole Abdomen	123	123	0	19	NA	17	11	23	72	60.7 [40.6, 78.5]	75.8 [65.9, 84.0]
**Prodhomme, 2015**	Gastro-intestinal	169	169	0	0	NA	15	10	0	144	60.0 [38.7, 78.9]	100 [97.4, 100]
**Tuchtan, 2018**	Gastro-intestinal	75	75	0	0	NA	2	0	1	72	66.7 [20.7, 93.9]	100 [94.9, 100]
**Prodhomme, 2015**	Urinary System	169	169	0	0	NA	37	2	0	130	94.9 [82.7, 99.4]	100 [97.2, 100]
**Tuchtan, 2018**	Urinary System	75	75	0	0	NA	9	1	0	65	90.0 [59.6, 98.2]	100 [94.4, 100]

Confidential: For Review Only

Two studies did not explicitly state the non-diagnostic imaging or non-diagnostic autopsies rate [18, 19]. Kang et al [17] reported a non-diagnostic ultrasound result for 13/70 (18.6%) brain, 21/123 (17.1%) thoracic, 24/122 (19.7%) cardiac and 19/123 (15.4%) abdominal examinations. The non-diagnostic autopsy rate was 53/123 (43.1%) for the brain and 1/123 (0.8%) for the heart.

Votino et al[20] reported non-diagnostic ultrasound imaging in 4/62 (6.5%) brain and 2/86 (2.3%) cardiothoracic ultrasound examinations. The non-diagnostic autopsy rate was 17/88 (19.3%) for the brain. In 5/88 (5.7%) cases, the brain was not examined at autopsy, only the body.

Four studies were included, all related to ultrasound usage in perinatal deaths. The pooled diagnostic sensitivity and specificity respectively was 85% (95% CI: 72%, 93%; $I^2 = 59\%$) and 97% (86%, 99%; $I^2 = 79\%$) for neurological abnormalities; 52% (26%, 78%, $I^2 = 79\%$) and 97% (87%, 99%; $I^2 = 81\%$) for cardiothoracic abnormalities and 80% (61%, 91%; $I^2 = 63\%$) and 98% (90%, 99%; $I^2 = 87\%$) for abdominal abnormalities. Combining all body systems, the pooled sensitivity was 74% (60%, 85%; $I^2 = 80\%$) and the pooled specificity was 97% (93%, 99%; $I^2 = 81\%$).

The study by Kang et al [14] appeared to be an outlier compared to the other three studies, particularly for specificity rates of all body systems. When excluding this outlier the pooled diagnostic sensitivity and specificity respectively was 88% (95% CI: 82%, 93%; $I^2 = 0\%$) and 98% (91%, 100%; $I^2 = 72\%$) for neurological abnormalities; 60% (22%, 89%, $I^2 = 84\%$) and 99% (95%, 100%; $I^2 = 63\%$) for cardiothoracic abnormalities and 84% (64%, 94%; $I^2 = 63\%$) and 99% (95%, 100%; $I^2 = 45\%$) for abdominal abnormalities. Combining all body systems, the pooled sensitivity was 81% (66%, 90%; $I^2 = 78\%$) and the pooled specificity was 98% (97%, 99%; $I^2 = 61\%$). See **Appendix, Figures S2 and S3** for the pooled sensitivity and specificity forest plots where this study has been excluded.

Confidential: For Review Only

DISCUSSION:

Post-mortem ultrasound for perinatal deaths is a feasible technique, and currently performed in a few European centres. The diagnostic accuracy rates are highest for neurological and abdominal abnormalities (pooled sensitivity rates of 85% and 82% respectively), but less effective for cardiothoracic abnormalities (pooled sensitivity rate of 49%). There were no studies reporting the post-mortem ultrasound accuracy rates in neonates and older children.

The study raises two main potential clinical implications, firstly relating to improved parental choice and secondly potential healthcare cost savings. It is well known that parents report a high acceptability for non-invasive autopsy methods [3, 26, 27], and that many healthcare professionals also advocate post-mortem imaging techniques [28]. Having another imaging modality available, such as ultrasound, would likely increase options for parental choice and their access to imaging. It may serve as a means for opening channels of communication between parents and clinicians, possibly reduce time spent grieving by offering a diagnosis and form of closure, or highlight the need for further counselling. As pregnant mothers will ubiquitously have been subject to fetal or antenatal ultrasound, they may have better inherent understanding of post-mortem ultrasound rather than cross-sectional imaging techniques.

In terms of finances, a previous systematic review by Ahmad et al [4] described a potential cost saving with MRI virtopsy compared to invasive autopsy of approximately 33% (based on a full body MRI costing £226.34 and an autopsy costing £471.80, with a paediatric MRI virtopsy sensitivity rate of 73%). Using their same reference for costing [29], an ultrasound study taking approximately 20 minutes (costing £56 per case, with similar whole body ultrasound pooled sensitivity rate of 74%), could offer even further benefits. Nevertheless, this calculation is based on assuming a similar rate of referral for invasive autopsy, which may be unlikely given the potential higher parental acceptability. Whether the balance between increased referrals and reduction in cost per patient would negate potential savings remains to be seen.

The comparatively low post-mortem ultrasound sensitivity rates for cardiothoracic abnormalities is also important to recognise, and may warrant alternative imaging with higher sensitivity (e,g, PMMR; 60% - 82% [30, 31]). Whether detection of abnormalities with ultrasound will improve with operator experience, or is related to the technique itself is unknown. The lack of circulation and inability to utilise Doppler imaging on ultrasound,

Confidential: For Review Only

teamed with the presence of post mortem intracardiac gas may hamper sonographic visualisation of cardiac structural abnormalities but 3D post-mortem ultrasound may possibly resolve some of these issues in future studies. In addition, PMUS operators may undergo a learning curve similar to that found for thoracic abnormalities using PMMR, with improvements in diagnostic accuracy from 30 – 37% in early reports [32] to 81.8%[31] later. This may relate to better knowledge of normal post-mortem changes on imaging (i.e. not overcalling pleural effusions as pathological) although infective pathologies still remain difficult to detect.

In this systematic review, the majority of studies reported similar ultrasound diagnostic accuracy rates although those by Kang et al [17] were much lower, particularly for sensitivity rates of neurological and thoracic abnormalities and, specificity rates for all body systems. Reasons for this are unclear, although the authors did report a high non-diagnostic ultrasound rate for brain imaging of approximately 13/70 (18.6%) and for thoracic imaging of 21/123 (17.1%). While demographic details of the perinatal deaths in their study were similar to others, the high non-diagnostic rate may suggest a greater referral of macerated fetuses, which could have impeded ultrasound visualisation of certain organs and thus accurate diagnosis. In addition, there was also a laparoscopic assisted minimally invasive autopsy as the reference standard in a subset of cases (11/123, 9%) [21]. Whilst this technique has been shown to provide a high success rate in terms of tissue sampling and detection of histological abnormalities, the true diagnostic accuracy rate compared to conventional autopsy remains unknown [33]. Despite these factors, we still include the results in our pooled analysis given that this was the only multicentre trial in our review and described results from more than one ultrasound operator, thereby providing potentially a more generalisable result for post-mortem ultrasound outcomes.

There were several limitations to this study. The first relates to the generalizability of our results. There were very few studies assessing the diagnostic accuracy of post-mortem ultrasound in children, with all studies focussing on perinatal deaths, conducted within Europe, and with ultrasound examinations being performed by the same operator within each centre. The examinations were conducted within a few specialist centres focussing on this niche area, meaning they may represent an elevated level of accuracy, potentially not achievable if post-mortem ultrasound was to be offered in a more general clinical setting.

The second limitation includes the classification and detection of abnormalities. In all cases the pathologists performing the autopsies were not blinded to the antenatal history, and in one of the studies the ultrasound

Confidential: For Review Only

operator was also unblinded. There may have been a variation in the way post-mortem ultrasounds are performed and reported. For example, some operators may have considered ascites and pleural effusions as part of normal post-mortem change [34, 35], whilst others may view these as significant abnormalities. There may also have been variation regarding the recording of non-diagnostic ultrasound examinations. In 2 studies the number of non-diagnostic autopsies and ultrasound examinations were not reported [18, 19], and it is not clear whether those ultrasound examinations were all of diagnostic quality, whether these were excluded prior to analysis, or whether the authors simply recorded these as 'no abnormality' where no significant findings were found, regardless of diagnostic quality. In the other two papers where non-diagnostic ultrasound examinations were reported, one excluded these from analysis [17] whereas the other considered them as an inaccurate finding (i.e. recorded as 'false positive')[20]. Nevertheless despite such limitations, our work has several strengths. This paper represents the first systematic review on use of post-mortem ultrasound in perinatal deaths. It demonstrates a reasonable diagnostic accuracy for the majority of body systems and that it can be performed on a variety of scanner models, in a varied cohort of perinatal deaths across a range of gestational ages.

Currently there are no standardized protocols on how best to perform a post-mortem ultrasound study, nor for which situations in would be most beneficial in helping to change clinical management for future pregnancies. For example, it may have an apparent higher diagnostic rate in fetal demise for those pregnancies who have not yet undergone detailed second or third trimester antenatal ultrasound (i.e after 20 weeks gestation).

Further research should be direct towards PMUS studies involving a larger cohort of perinatal deaths and also assessing usage in older children. There is clearly potential for this technique to be adopted in different clinical settings (such as in the developing world where pathological expertise is lacking but where causes of death are still important to confirm to inform global public health initiatives [36]), by other specialists to increase appeal and availability following adequate training (e.g. pathologists, obstetricians, sonographers) and international guidelines should be established for the appropriate indications for usage. There is also the ability for ultrasound to be used as a tool to aid percutaneous organ biopsies where tissue sampling is required to confirm histological diagnoses. In conclusion, this systematic review has provided evidence in showing that it is a feasible technique with a reasonable diagnostic accuracy when performed by experienced operators, however further work will enable a better understanding of its place and purpose within a future non-invasive autopsy service.

Confidential: For Review Only

ACKNOWLEDGEMENTS:

1. **Conflict of Interest:**

All authors have completed the ICMJE uniform disclosure forms and declare: no support from any organisation for the submitted work; no financial relationships with any organisations that might have an interest in the submitted work in the previous three years; no other relationships or activities that could appear to have influenced the submitted work.

2. **Role of Funding Source**

The funder of the study had no role in the study design, data collection, data analysis, data interpretation, or writing of the report. The corresponding author and chief investigator had full access to all the data in the study and final responsibility for decision to submit for publication.

3. **Ethical Approval:**

Institutional review board approval was not required because it comprises a systematic review of published literature.

4. **Informed Consent:**

Written informed consent was not required for this study because it comprises a systematic review of published literature.

5. **Funding**

SCS is supported by a RCUK/ UKRI Innovation Fellowship and Medical Research Council (MRC) Clinical Research Training Fellowship (Grant Ref: MR/R00218/1). This award is jointly funded by the Royal College of Radiologists (RCR). OJA is funded by a National Institute for Health Research (NIHR) Career Development Fellowship (NIHR-CDF-2017-10-037), and NJS funded by an NIHR Senior Investigator award.

The authors receive funding from the Great Ormond Street Children's Charity and the Great Ormond Street Hospital NIHR Biomedical Research Centre. This article presents independent research funded by

Confidential: For Review Only

the MRC, RCR, NIHR and the views expressed are those of the author(s) and not necessarily those of the NHS, MRC, RCR, the NIHR or the Department of Health.

6. Author Contribution

All authors listed in this manuscript fulfil the ICMJE recommendations for authorship. All authors provided substantial contribution to the conception and design of the work, analysis and interpretation; and drafting the work for intellectual content. All authors have had final approval for the version to be published and agree to be accountable for all aspects of the work in ensuring that questions related to the accuracy are appropriately investigated and resolved.

7. Data Sharing Statement

All data is provided in full in the results section of this paper and in the appendices for search terminologies. Raw numbers provided in an excel spreadsheet are available upon request from the main author.

Confidential: For Review Only

FIGURE LEGENDS:

Figure 1

PRISMA flow chart for the study search and selection

Figure 2

Methodological quality assessment of the included studies using the QUADAS-2 tool. Risk of bias and applicability concerns summary about each domain are shown for each included study

Figure 3

Pooled sensitivity forest plots including 95% confidence intervals (CI) for all studies, grouped by body systems.

Figure 4

Pooled specificity forest plots including 95% confidence intervals (CI) for all studies, grouped by body systems.

Confidential: For Review Only

REFERENCES:

1. Sieswerda-Hoogendoorn T, van Rijn RR. Current techniques in postmortem imaging with specific attention to paediatric applications. *Pediatr Radiol* 2010; **40**: 141-152; quiz 259. DOI: 10.1007/s00247-009-1486-0.

2. Arthurs OJ, van Rijn RR, Sebire NJ. Current status of paediatric post-mortem imaging: an ESPR questionnaire-based survey. *Pediatr Radiol* 2014; **44**: 244-251. DOI: 10.1007/s00247-013-2827-6.

3. Lewis C, Hill M, Arthurs OJ, Hutchinson C, Chitty LS, Sebire NJ. Factors affecting uptake of postmortem examination in the prenatal, perinatal and paediatric setting. *Bjog* 2018; **125**: 172-181. DOI: 10.1002/uog.17468 10.1111/1471-0528.14600.

4. Ahmad MU, Sharif KA, Qayyum H, Ehsanullah B, Balyasnikova S, Wale A, Shanmuganandan A, Siddiqui MRS, Athanasiou T, Kemp GJ. Assessing the use of magnetic resonance imaging virtopsy as an alternative to autopsy: a systematic review and meta-analysis. *Postgrad Med J* 2017; **93**: 671-678. DOI: 10.1136/postgradmedj-2017-134945.

5. van Rijn RR, Beek EJ, van de Putte EM, Teeuw AH, Nikkels PGJ, Duijst W, Nievelstein RA, Dutch NG. The value of postmortem computed tomography in paediatric natural cause of death: a Dutch observational study. *Pediatr Radiol* 2017; **47**: 1514-1522. DOI: 10.1007/s00247-017-3911-0.

6. Arthurs OJ, Guy A, Thayyil S, Wade A, Jones R, Norman W, Scott R, Robertson NJ, Jacques TS, Chong WK, Gunny R, Saunders D, Olsen OE, Owens CM, Offiah AC, Chitty LS, Taylor AM, Sebire NJ. Comparison of diagnostic performance for perinatal and paediatric post-mortem imaging: CT versus MRI. *Eur Radiol* 2016; **26**: 2327-2336. DOI: 10.1007/s00330-015-4057-9.

7. Oyake Y, Aoki T, Shiotani S, Kohno M, Ohashi N, Akutsu H, Yamazaki K. Postmortem computed tomography for detecting causes of sudden death in infants and children: retrospective review of cases. *Radiat Med* 2006; **24**: 493-502. DOI: 10.1007/s11604-006-0061-y.

8. Sieswerda-Hoogendoorn T, Soerdjbalie-Maikoe V, de Bakker H, van Rijn RR. Postmortem CT compared to autopsy in children; concordance in a forensic setting. *Int J Legal Med* 2014; **128**: 957-965. DOI: 10.1007/s00414-014-0964-6.

9. Shelmerdine SC, Langan D, Hutchinson JC, Hickson M, Pawley K, Suich J, Palm L, Sebire NJ, Wade A, Arthurs OJ. Chest radiographs versus CT for the detection of rib fractures in children (DRIFT): a diagnostic accuracy observational study. *The Lancet Child & adolescent health* 2018; **2**: 802-811. DOI: 10.1016/s2352-4642(18)30274-8.

10. Prodhomme O, Baud C, Saguintaah M, Béchard-Sevette N, Bolivar J, David S, Taleb-Arrada I, Couture A. Principles of fetal postmortem ultrasound: A personal review. *Journal of Forensic Radiology and Imaging* 2015; **3**: 12-15. DOI: 10.1016/j.jofri.2015.01.008.

11. Kumar M, Priyam. Ultrasonography and autopsy correlation of fetal hypoplastic left heart syndrome. *Journal of clinical ultrasound : JCU* 2018. DOI: 10.1002/jcu.22607.

12. Shelmerdine SC. Diagnostic accuracy of paediatric post-mortem ultrasound compared with autopsy: a systematic review. https://www.crd.york.ac.uk/prospero/display_record.php?RecordID=106968 [1 Sept 2018]

13. Moher D, Liberati A, Tetzlaff J, Altman DG, Group P. Preferred reporting items for systematic reviews and meta-analyses: the PRISMA statement. *BMJ (Clinical research ed)* 2009; **339**: b2535. DOI: 10.1136/bmj.b2535.

14. Whiting PF, Rutjes AW, Westwood ME, Mallett S, Deeks JJ, Reitsma JB, Leeflang MM, Sterne JA, Bossuyt PM. QUADAS-2: a revised tool for the quality assessment of diagnostic accuracy studies. *Annals of internal medicine* 2011; **155**: 529-536. DOI: 10.7326/0003-4819-155-8-201110180-00009.

Confidential: For Review Only

15. Team RC. R: A language and environment for statistical computing. R Foundation for Statistical Computing: Vienna, Austria, 2016.

16. Schwarzer G. Meta: An R package for meta-analysis. *R News* 2007; **7**: 40-45.

17. Kang X, Shelmerdine SC, Hurtado I, Bevilacqua E, Hutchinson C, Mandalia U, Segers V, Cos Sanchez T, Cannie MM, Carlin A, Sebire NJ, Arthurs OJ, Jani JC. Postmortem examination of human fetuses: a comparison of 2-dimensional ultrasound with invasive autopsy. *Ultrasound Obstet Gynecol* 2017. DOI: 10.1002/uog.18828.

18. Prodhomme O, Baud C, Saguintaah M, Béchard-Sevette N, Bolivar J, David S, Taleb-Arrada I, Couture A. Comparison of postmortem ultrasound and X-Ray with autopsy in fetal death: Retrospective study of 169 cases. *Journal of Forensic Radiology and Imaging* 2015; **3**: 120-130. DOI: 10.1016/j.jofri.2015.04.002.

19. Tuchtan L, Lesieur E, Bartoli C, Delteil C, Sarda-Quarello L, Torrents J, Sigaudy S, Piercecchi MD, Gorincour G. Diagnosis of congenital abnormalities with post-mortem ultrasound in perinatal death. *Diagnostic and interventional imaging* 2018; **99**: 143-149. DOI: 10.1016/j.diii.2017.11.005.

20. Votino C, Cos Sanchez T, Bessieres B, Segers V, Kadhim H, Razavi F, Condorelli M, Votino R, D'Ambrosio V, Jani J. Minimally invasive fetal autopsy using ultrasound: a feasibility study. *Ultrasound in Obstetrics and Gynecology* 2018; **52**: 776-783.

21. Sebire NJ, Weber MA, Thayyil S, Mushtaq I, Taylor A, Chitty LS. Minimally invasive perinatal autopsies using magnetic resonance imaging and endoscopic postmortem examination ("keyhole autopsy"): feasibility and initial experience. *J Matern Fetal Neonatal Med* 2012; **25**: 513-518. DOI: 10.3109/14767058.2011.601368.

22. Royal College of Pathologists. Guidelines on autopsy practice: Fetal autopsy (2nd trimester fetal loss and termination of pregnancy for congenital anomaly). www.rcpath.org [Accessed 16 May 2018].

23. Royal College of Pathologists. Guidelines on autopsy practice: Third trimester antepartum and intrapartum stillbirth. www.rcpath.org [Accessed 16 May 2018].

24. Societe Francaise de Foetopathologie. http://soffoet.fr/page-d-exemple/la-foetopathologie/ [Accessed 09 January 2019].

25. Haute Autorite de Sante. https://www.has-sante.fr/portail/jcms/r_1455081/fr/home-page?portal=r_1455081 [Accessed 9th January 2019].

26. Kang X, Cos T, Guizani M, Cannie MM, Segers V, Jani JC. Parental acceptance of minimally invasive fetal and neonatal autopsy compared with conventional autopsy. *Prenat Diagn* 2014; **34**: 1106-1110. DOI: 10.1002/pd.4435.

27. Cannie M, Votino C, Moerman P, Vanheste R, Segers V, Van Berkel K, Hanssens M, Kang X, Cos T, Kir M, Balepa L, Divano L, Foulon W, De Mey J, Jani J. Acceptance, reliability and confidence of diagnosis of fetal and neonatal virtuopsy compared with conventional autopsy: a prospective study. *Ultrasound Obstet Gynecol* 2012; **39**: 659-665. DOI: 10.1002/uog.10079.

28. Lewis C, Hill M, Arthurs OJ, Hutchinson JC, Chitty LS, Sebire N. Health professionals' and coroners' views on less invasive perinatal and paediatric autopsy: a qualitative study. *Archives of disease in childhood* 2018. DOI: 10.1136/archdischild-2017-314424.

29. NHS National Tariff Payment System 2016/17. Annex A. https://www.gov.uk/government/publications/nhs-national-tariff-payment-system-201617 [Accessed 24 January 2019].

30. Taylor AM, Sebire NJ, Ashworth MT, Schievano S, Scott RJ, Wade A, Chitty LS, Robertson N, Thayyil S. Postmortem cardiovascular magnetic resonance imaging in fetuses and children: a masked

Confidential: For Review Only

comparison study with conventional autopsy. *Circulation* 2014; **129**: 1937-1944. DOI: 10.1161/circulationaha.113.005641.

31. Ashwin C, Hutchinson JC, Kang X, Langan D, Jones R, Norman W, Cannie M, Jani J, Sebire NJ, Arthurs OJ. Learning effect on perinatal post-mortem magnetic resonance imaging reporting: single reporter diagnostic accuracy of 200 cases. *Prenat Diagn* 2017; **37**: 566-574. DOI: 10.1002/pd.5043.

32. Arthurs OJ, Thayyil S, Olsen OE, Addison S, Wade A, Jones R, Norman W, Scott RJ, Robertson NJ, Taylor AM, Chitty LS, Sebire NJ, Owens CM. Diagnostic accuracy of post-mortem MRI for thoracic abnormalities in fetuses and children. *Eur Radiol* 2014; **24**: 2876-2884. DOI: 10.1007/s00330-014-3313-8.

33. Hutchinson C, Shelmerdine SC. Feasibility and experience of the MinImAL procedure: Minimally Invasive perinatal and paediatric Autopsies with Laparoscopically assisted tissue sampling. 2019. DOI: 10.1002/uog.20211.

34. Arthurs OJ, Barber JL, Taylor AM, Sebire NJ. Normal perinatal and paediatric postmortem magnetic resonance imaging appearances. *Pediatr Radiol* 2015; **45**: 527-535. DOI: 10.1007/s00247-014-3166-y.

35. Barber JL, Hutchinson JC, Sebire NJ, Arthurs OJ. Pleural fluid accumulation detectable on paediatric post-mortem imaging: a possible marker of interval since death? 2016; **130**: 1003-1010. DOI: 10.1007/s00414-016-1320-9.

36. Menendez C, Castillo P, Martinez MJ, Jordao D, Lovane L, Ismail MR, Carrilho C, Lorenzoni C, Fernandes F, Nhampossa T, Hurtado JC, Navarro M, Casas I, Santos Ritchie P, Bandeira S, Mocumbi S, Jaze Z, Mabota F, Munguambe K, Maixenchs M, Sanz A, Mandomando I, Nadal A, Gonce A, Munoz-Almagro C, Quinto L, Vila J, Macete E, Alonso P, Ordi J, Bassat Q. Validity of a minimally invasive autopsy for cause of death determination in stillborn babies and neonates in Mozambique: An observational study. *PLoS Med* 2017; **14**: e1002318. DOI: 10.1371/journal.pmed.1002318.

Confidential: For Review Only

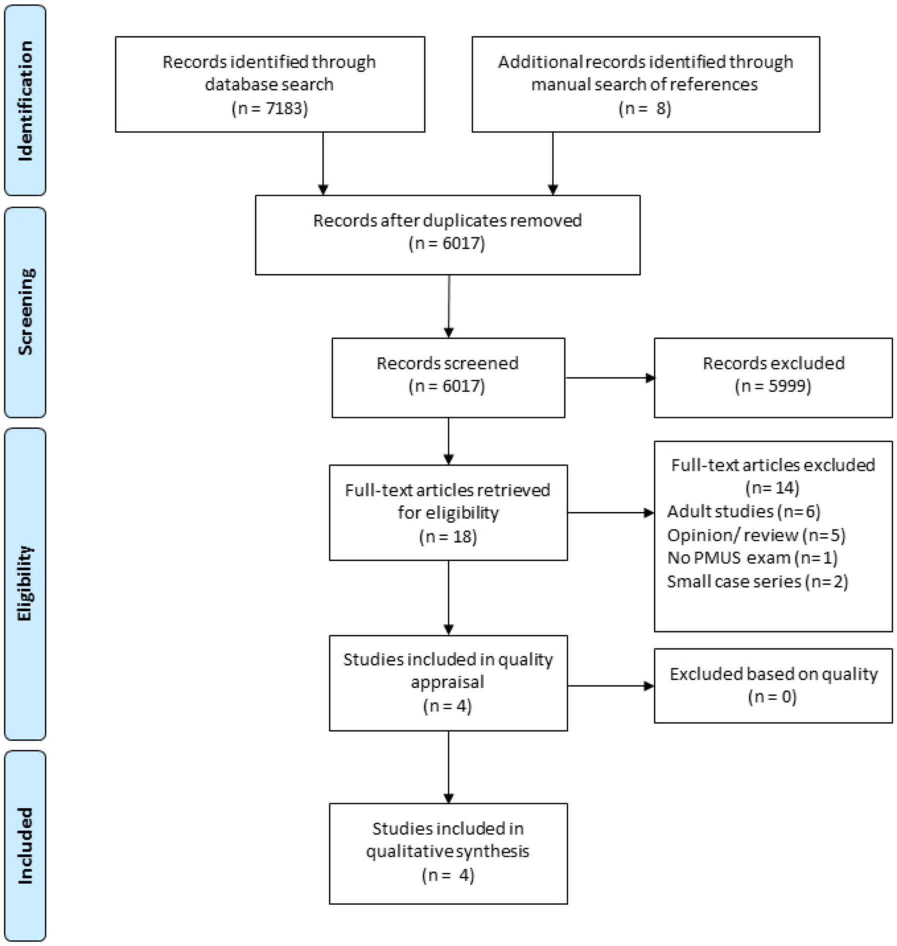

200x190mm (96 x 96 DPI)

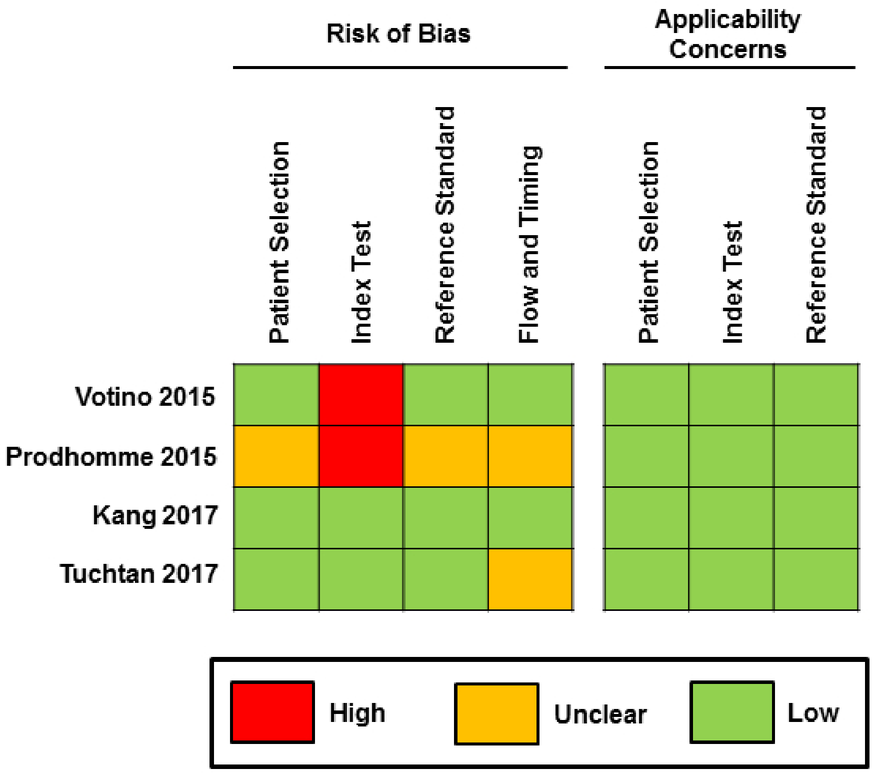

167x149mm (96 x 96 DPI)

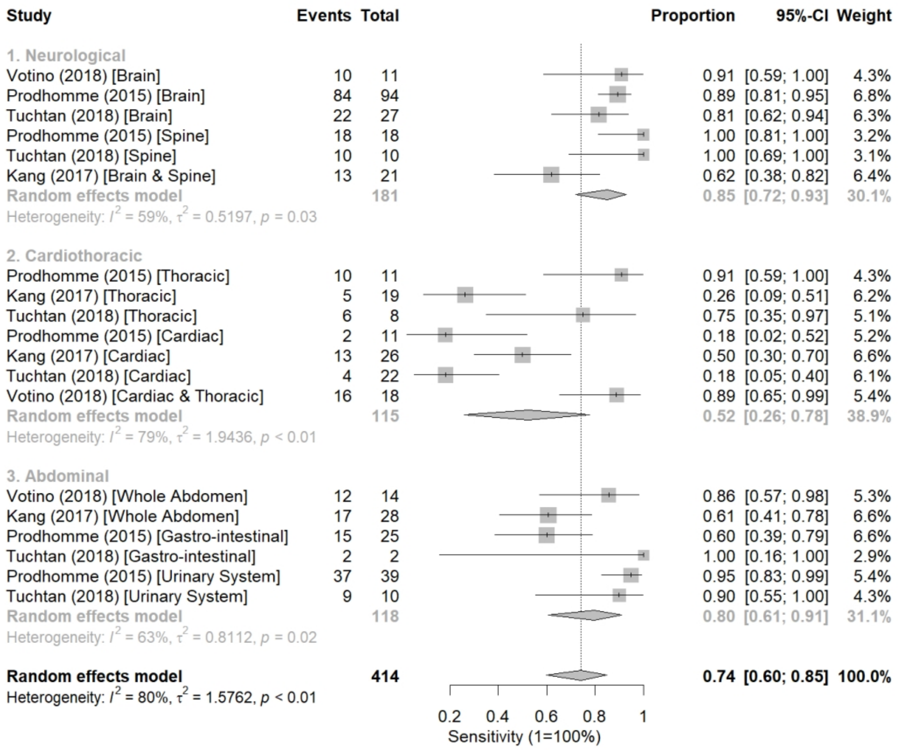

127x101mm (300 x 300 DPI)

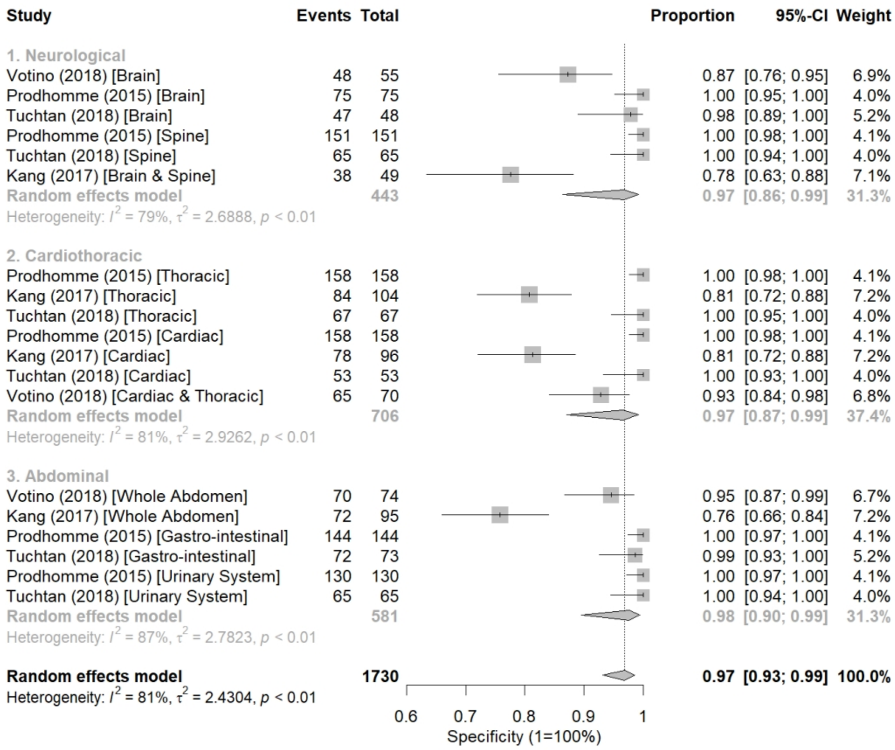

127x101mm (300 x 300 DPI)

Appendix

S1. Search strategy for retrieval of included studies

MEDLINE:

#	01 January 1998 to 31 December 2018	Results (n)
1	(Autopsy [Mesh] OR autopsy [tiab] OR necropsy [tiab] OR post-mortem [tiab] OR postmortem [tiab])	148316
2	(Infant death, sudden [Mesh] OR infant* [tiab] OR child*[tiab] OR adolescent [tiab] OR pediatric*[tiab] OR paediatric*[tiab] OR neonate*[tiab] OR newborn*[tiab] OR baby [tiab] OR babies [tiab] OR youth*[tiab] OR perinatal OR fetal [tiab] OR foetal [tiab] OR termination* [tiab] OR abortion* [tiab])	2276140
3	("Ultrasonography"[Mesh] OR ultraso*[tiab] OR sonograph*[tiab] OR echo*[tiab] OR imaging [tiab] OR radiology [tiab])	1308153
**4**	1 and 2 and 3	**3133**

EMBASE:

#	01 January 1998 to 31 December 2018; Human subjects only	Results (n)
1	Autopsy.a.f	68338
2	(postmortem or post-mortem).a.f	33839
3	1 or 2	87723
4	(child* or preschool child* or adolescent or infant* or newborn* or pediatric* or infant* or newborn* or neonat* or baby or babies or paediatric* or adoles* or teen* or youth* or fetal or foetal).a.f.	2900920
5	(ultraso* or sonograph* or echo*).a.f.	778476
**6**	**3 and 4 and 5**	**3915**

COCHRANE LIBRARY

#	01 January 1998 to 31 December 2018	Results (n)
1	Autopsy OR postmortem OR post-mortem (Word variations have been searched)	1181
2	child OR adolescent OR infant OR newborn OR pediatric OR neonate OR baby OR babies OR paediatric OR teen OR youth OR fetal OR foetal (Word variations have been searched)	240101
3	Ultrasonography OR ultraso* OR sonograph* OR echo* OR imaging OR radiology (Word variations have been searched)	102777
**4**	**#1 and #2 and #3**	**135**

Confidential: For Review Only

S2. Pooled sensitivity forest plots including 95% confidence intervals (CI) for all studies, grouped by body systems (excluding publication by Kang et al, 2017)

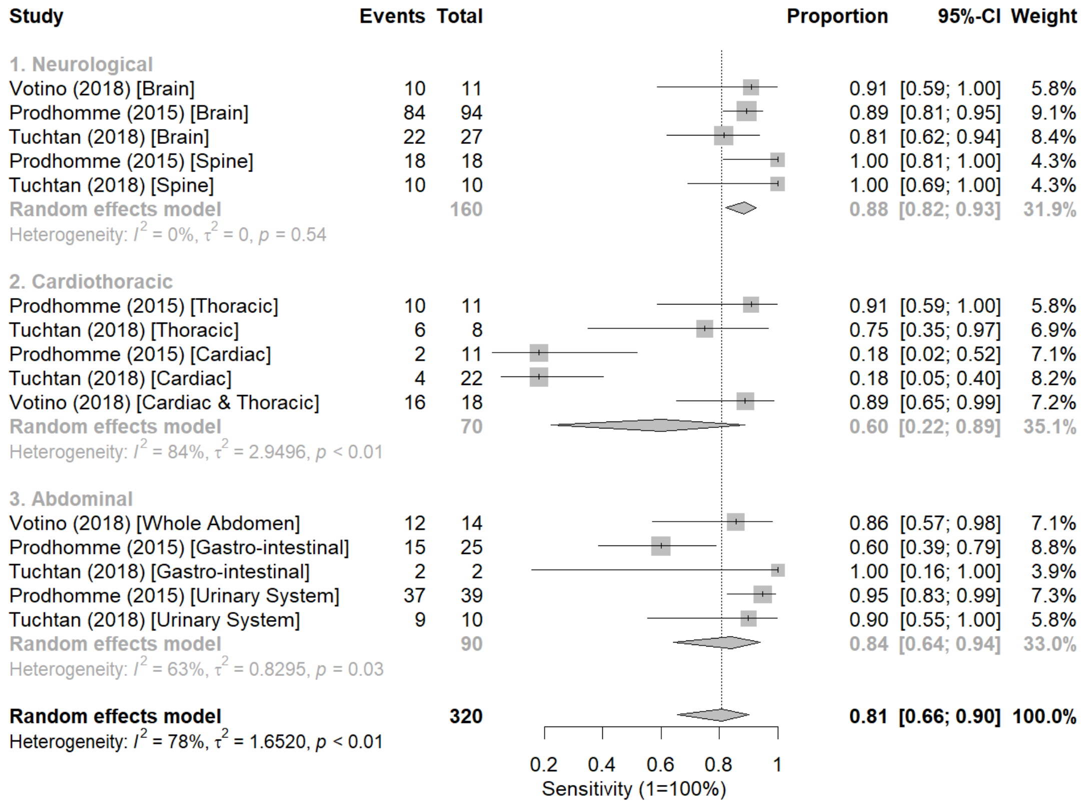

Review Only

S3. Pooled specificity forest plots including 95% confidence intervals (CI) for all studies, grouped by body systems (excluding Kang et al, 2017)

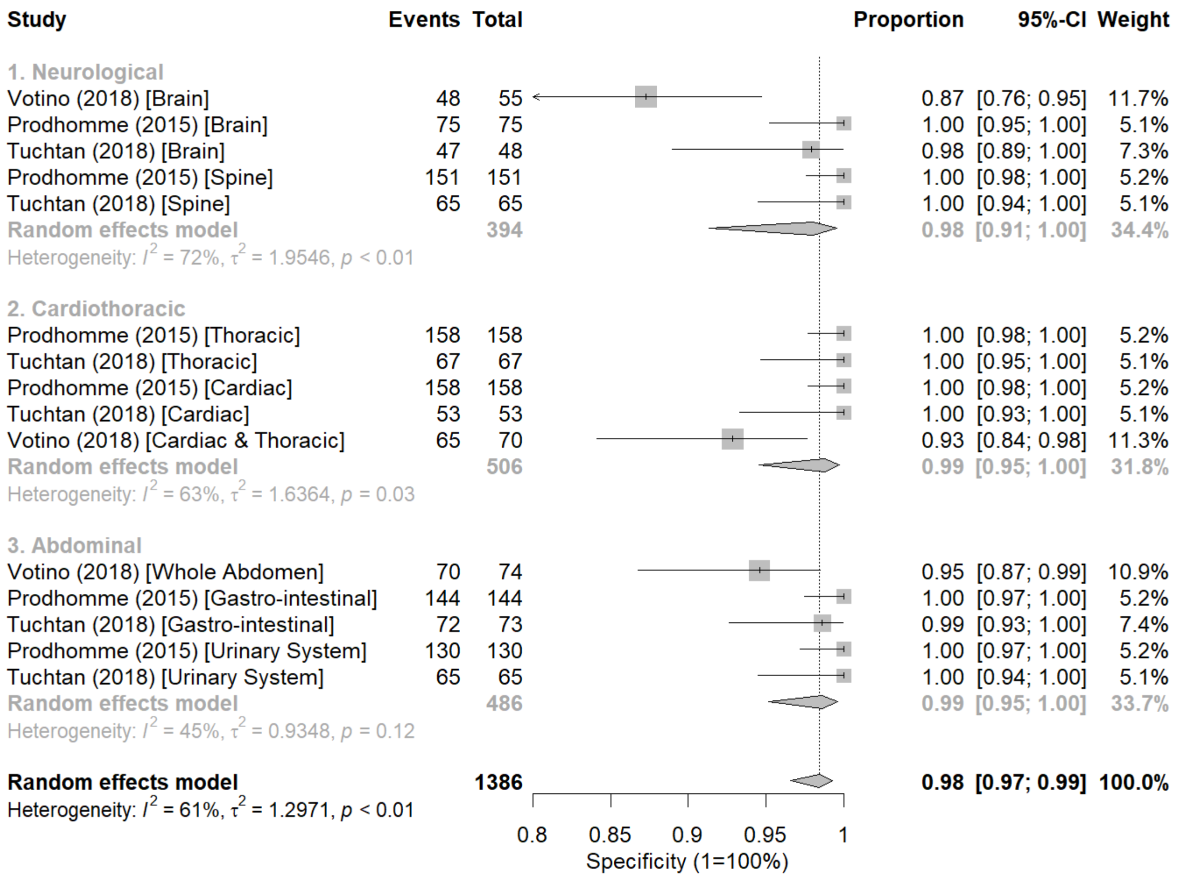

eview Only

BMJ Paediatrics Open

Diagnostic Accuracy of Perinatal Post-mortem Ultrasound (PMUS): A Systematic Review

Journal:	*BMJ Paediatrics Open*
Manuscript ID	bmjpo-2019-000566.R1
Article Type:	Original research
Date Submitted by the Author:	08-Oct-2019

Complete List of Authors:	Shelmerdine, Susan; Great Ormond Street Hospital for Children, Clinical Radiology; UCL GOSH ICH, Radiology<br>Langan, Dean; UCL GOSH ICH<br>Sebire, Neil; Great Ormond Street Hospital for Children, Paediatric Pathology; UCL GOSH ICH<br>Arthurs, Owen; Great Ormond Street Hospital for Children, Clinical Radiology; UCL GOSH ICH

Keywords:	Imaging, Pathology, General Paediatrics, Fetal Medicine, Evidence Based Medicine

SCHOLARONE™ Manuscripts

Title Page

Full Title:

Diagnostic Accuracy of Perinatal Post-mortem Ultrasound (PMUS): A Systematic Review

Short Title:

Diagnostic Accuracy of Post-mortem Ultrasound

Authors:

Susan Cheng Shelmerdine, MBBS, FRCR [a, b] ; susan.shelmerdine@gosh.nhs.uk

Dean Langan, PhD [b, c] ; d.langan@ucl.ac.uk

Neil James Sebire, Prof, MD [b, d] ; neil.sebire@gosh.nhs.uk

Owen John Arthurs, PhD [a, b] ; owen.arthurs@gosh.nhs.uk

Affiliations:

a Department of Clinical Radiology, Great Ormond Street Hospital for Children, London, UK

b UCL Great Ormond Street Institute of Child Health, Great Ormond Street Hospital for Children, London, UK.

c Centre of Applied Statistics Courses, University College London, London, UK

d Department of Paediatric Pathology, Great Ormond Street Hospital for Children, London, UK

Corresponding Author:

Name: Dr. Susan Shelmerdine

Job title: Paediatric Radiology Research Fellow

Address: Department of Clinical Radiology,

Great Ormond Street Hospital for Children, London, United Kingdom

Email: susan.shelmerdine@gosh.nhs.uk

Telephone: +44 (0) 20 9200 (x6369)

Fax: Not applicable.

Key words

Autopsy, minimally invasive autopsy, paediatric, perinatal, ultrasound

Word Count: 3223 words **Reference Count:** 36 references

Confidential: For Review Only

What is known about the subject:

- Whole body post-mortem ultrasound is being performed in several European centres for perinatal deaths.
- Perinatal post-mortem ultrasound can be a useful alternative technique for non-invasive autopsy, where MRI is unavailable.

What this study adds:

- Perinatal post-mortem ultrasound provides a high diagnostic accuracy for neurological and abdominal pathologies.
- Assessment of perinatal post-mortem cardiothoracic anomalies is challenging and less accurate.

Confidential: For Review Only

ABSTRACT:

Abstract:

Objective:

Ultrasound is ubiquitous in live paediatric imaging, however its usage in post-mortem setting is less established. This systematic review aims to evaluate the diagnostic accuracy of paediatric post-mortem ultrasound, PMUS.

Design:

MEDLINE, Embase and Cochrane Library databases were queried for studies published between 1998 – 2018 assessing PMUS diagnostic accuracy rates in children <18 years old, using autopsy as reference standard. Risk of bias was assessed using QUADAS 2. A bi-variate random-effects model was used to obtain combined mean estimates of sensitivity and specificity for different body systems.

Results:

Four studies were included, all relating to ultrasound for perinatal deaths. The mean diagnostic sensitivity and specificity for neurological abnormalities was 84.3% (95% CI: 70.8%, 92.2%) and 96.7% (95% CI: 86.5%, 99.3%); for cardiothoracic abnormalities 52.1% (95% CI: 27.6%, 75.5%,) and 96.6% (95% CI: 86.8%, 99.2%); and for abdominal abnormalities 78.4% (95% CI: 61.0%, 89.4%) and 97.3% (95% CI: 88.9%, 99.4%). Combining all body systems, the mean sensitivity and specificity was 73.3% (95% CI: 59.9%, 83.5%) and 96.6% (95% CI: 92.6%, 98.4%).

Conclusions:

PMUS demonstrates a reasonable diagnostic accuracy, particularly for abdominal and neurological abnormalities, although cardiac anomalies were less readily identified.

Trial registration:

This study was registered in PROSPERO International prospective register of systematic reviews, CRD42018106968.

KEYWORDS:

Autopsy, minimally invasive autopsy, paediatric, perinatal, ultrasound

Confidential: For Review Only

ABBREVIATIONS:

PRISMA	Preferred Reported Items for Systematic Reviews and Meta-Analysis statement
PROSPERO	International Prospective Register of Systematic Reviews
PMUS	Post-mortem ultrasound
PMMR	Post-mortem magnetic resonance imaging
PMI	Post-mortem interval (i.e. time between death and imaging)

INTRODUCTION:

Post-mortem imaging techniques in children have gained popularity over the past decade, particularly given the decline in parental consent rates for invasive autopsy [1, 2]. Factors for this are multifaceted but include religious, emotional and communication barriers combined with the continued desire for further information to aid in future pregnancy management and bereavement counselling [3].

The majority of studies relating to post-mortem imaging in children have related to post-mortem MRI (PMMR), with a recently published systematic review [4] reporting sensitivity rates of 0.73 (95% CI 0.56 – 0.84) in a pooled sample of 953 children across eight studies. For post-mortem CT imaging (PMCT), there have been fewer studies in children compared to adults, with the majority reporting CT to be of lower yield in identification for the cause of death in non-suspicious (i.e. 'natural' deaths) and perinatal losses [5-7], with a slight increase in benefit within a forensic setting [8, 9].

Ultrasound, whilst ubiquitous in live paediatric imaging, is far less established in the post-mortem setting. There are only a few articles either describing single site experience [10], or focussing on single anatomical areas such as congenital cardiac disorders [11]. With rising costs, radiographer staffing shortages and reduced availability and access to MRI and CT imaging, ultrasound may play a key role in a future non-invasive post-mortem imaging service. The equipment is cheaper to acquire and run than cross-sectional imaging, with a variety of healthcare specialists already skilled in sonographic techniques (e.g. fetal medicine clinicians, obstetricians, radiologists, sonographers). Nevertheless, without robust evidence detailing the diagnostic accuracy of this technique, it remains difficult to counsel parents appropriately and for clinicians to understand the benefit of this modality as a non-invasive imaging alternative to conventional autopsy. The aim of this systematic review is therefore to

Confidential: For Review Only

comprehensively assess the diagnostic accuracy of paediatric post-mortem ultrasonography (PMUS) from the existing literature.

MATERIALS AND METHODS:

This study was registered in PROSPERO International prospective register of systematic reviews, CRD42018106968[12]. PRISMA (Preferred Reporting Items for Systematic reviews and Meta-Analyses) guidelines for transparent reporting of systematic reviews were followed [13]. Ethical approval was not required for this retrospective review of published data.

Patient Involvement

Patients were not directly involved in the design of this study.

Literature Review

We searched MEDLINE (Ovid), EMBASE and the Cochrane Library databases for eligible articles published between 1 January 1998 and 31 December 2018 (20 year range), using database specific Boolean search strategies with terms and word variations relating to 'autopsy', 'ultrasonography' and 'paediatrics'. The full search strategy was conducted in December 2018 (See Appendix S1 for details). A repeat search was conducted in July 2019 did not reveal any further eligible manuscripts for inclusion.

Eligibility Criteria

Inclusion criteria encompassed work investigating diagnostic accuracy of post-mortem ultrasound imaging using autopsy as a reference standard. Studies were limited to human subjects, including fetuses (any gestation) and children (ages 0- 18 years). No restrictions were placed on method of ultrasound technique, machine vendor, and experience of operator or type of clinical setting. No language restrictions were used.

Exclusion criteria included studies reported as conference abstracts, case reports, editorials, opinion articles, pictorial reviews, multimedia files (online videos, podcasts) and small case series (fewer than 5 cases). Articles without autopsy reference standard, or those relating to diagnostic accuracy for antenatal ultrasound imaging were excluded.

https://mc.manuscriptcentral.com/bmjpo

Confidential: For Review Only

All articles were independently searched by two reviewers (both paediatric radiologists with >8 years (SCS) and >12 years (OJA) experience). Abstracts of suitable studies were examined, and full papers were obtained. References from the retrieved full text articles were manually examined for other possible publications. Disagreements were resolved by consensus.

Data extraction and Quantitative Data Synthesis

Two reviewers (SCS, OJA) independently extracted data from the full articles into a database (Excel, Microsoft, Redmond WA, USA) which included the following factors: study design, study setting, population demographics, sample size, index test, study methodology, use of reference standard, diagnostic accuracy outcomes, measure of bias.

Raw numbers of true and false positives and negative post-mortem ultrasound diagnoses were collected and inputted into a 2 x 2 table, considering autopsy as reference standard. Missing data were calculated if possible and, unpublished data or further information was obtained by contacting the authors.

Methodological Quality

The quality and measure of bias for each included study was assessed by the modified Quality Assessment of Diagnostic Accuracy Studies (QUADAS 2) criteria [14]. Disagreements were resolved by consensus review of the literature.

Statistical Analysis

Statistical analysis was performed by an experienced statistician (DL) using R [15]. The available data was used to compute sensitivity and specificity for each study, with a 0.5 correction factor for studies with zero frequencies. A bi-variate random-effects model was used to obtain combined mean estimates of sensitivity and specificity with 95% confidence intervals for overall PMUS diagnosis and, by three body systems (neurological, cardiothoracic and abdominal). The functions *madad* and *reitsma* within the R package *mada* was used to synthesise the evidence. This bivariate approach takes into account the correlation between sensitivity and specificity when different threshold values are used for diagnosis in each study/body system [16]. SROC curves were presented to show the relationship between the two outcomes. Spearman's correlation coefficient (rho) was calculated to test for the presence of different thresholds.

Confidential: For Review Only

RESULTS:

Eligible Studies

The initial search performed on 1 January 2019 yielded 6017 articles, after removal of duplicate studies. On the basis of study title and abstract, 5999 articles were excluded and 18 articles were retrieved in full text. After review of the full text, four studies were eventually included in the systematic review [17-20]. A PRISMA flowchart is shown in **Figure 1**.

Patient Population

Our patient and study characteristics are provided in **Tables 1 and 2**. All four articles related to post-mortem ultrasound for perinatal deaths [17-20]. In all studies, parental consent was given for the ultrasound imaging. In total, 495 fetuses were imaged (sample size range: 75 - 169) and of these, 455 underwent an autopsy as a reference standard. The gestational ages ranged from 11 – 48 weeks. Most of the fetuses were the result of terminations of pregnancy (363/495, 73%), with 11% (52/495) miscarriages and 16% (80/495) intrauterine deaths. Gender mix was not reported in any of the studies.

Confidential: For Review Only

Table 1: Demographic details of population studied for all articles

Author, year	Country	Sample Size, n	Patient Group	Median gestation at death	Gestational age range (weeks)	Median post-mortem weight (g)	Weight range (g)	Mode of Death
**Prodhomme, 2015**	France	169	Fetuses	27 weeks	15 – 38	Not stated	Not stated	97% (164) terminations<br>3% (5) intrauterine deaths
**Tuchtan, 2018**	France	75	Fetuses	Not stated, however 42 cases were <24 weeks; 33 were >24 weeks	15 – 38	Not stated	Not stated	79% (59) terminations<br>4% (3) miscarriages<br>17% (13) intrauterine deaths
**Votino, 2018**	Belgium	88	Fetuses	21 weeks	11 – 40	702	7 – 4020	66% (58) terminations<br>17% (15) miscarriages<br>17% (15) intrauterine deaths
**Kang, 2019**	Belgium & U.K.	163 imaged, 123 with reference test	Fetuses	23 weeks	13 – 42	Not stated	Not stated	50% (82) terminations<br>21% (34) miscarriages<br>29% (47) intrauterine deaths

Confidential: For Review Only

Table 2: Study characteristics for articles included in systematic review

Author, year	Country	Sample Size	Study Design	Number of Centres	Patient Selection	Study Period	Index Test	Reference Test
**Prodhomme, 2015**	France	169	Retrospective	Single	Unclear	4 years (2009 – 13)	2D whole body ultrasound	Conventional autopsy
**Tuchtan, 2018**	France	75	Prospective	Single	Consecutive	1 year (2014)	2D whole body ultrasound	Conventional autopsy
**Votino, 2018**	Belgium	88	Prospective	Single	Consecutive	19 months (2012 – 13)	2D and 3D whole body ultrasound	Conventional autopsy
**Kang, 2019**	Belgium & U.K.	163	Prospective	Multiple, 2 centres	Consecutive	2 years (2014-16)	2D whole body ultrasound	Conventional and minimally invasive autopsy

Confidential: For Review Only

The majority of studies (3/4, 75%) were prospective in design and conducted in a single centre (3/4, 75%). Two studies originated from France [18, 19], one study originated from Belgium [20] and one was a multicentre study involving patients from two institutions, one in the United Kingdom and one in Belgium [17]. The timeframe for data collection ranged between 1 – 4years.

Imaging Assessment and Operator

Details regarding the index (post-mortem ultrasound) and reference (autopsy) tests in the studies of this systematic review are summarised in **Tables 3 and 4**.

Confidential: For Review Only

Table 3: Details of index test for studies included for systematic review.

Author, year	Ultrasound Machine	Ultrasound Transducers/ Probes	Imaging time (mins)	Patient preparation	Ultrasound Operator, Experience	No. operators	Blinded to clinical history	Time between delivery to imaging	Diagnostic accuracy subgroup measures
**Prodhomme, 2015**	Phlips iU22	5-8 MHz microconvex<br>5-12 MHz linear<br>5-17 MHz linear	Not stated	No additional preparation over cold storage of body	Paediatric radiologist, >10 years' experience	Single	No	Not stated	Agreement with final autopsy diagnosis. List of diagnoses given.
**Tuchtan, 2018**	Toshiba Aplio 500; Supersonic Aixplorer;<br><br>GE Voluson E8	Toshiba:<br>7-10MHz curved<br>10-15MHz linear<br><br>Supersonic:<br>6-10MHz curved<br>11-15MHz linear<br><br>GE:<br>5-10MHz curved<br>10-14MHz linear	20	No additional preparation over cold storage of body	Paediatric Radiologist, 15years ultrasound and 8 years post-mortem imaging experience	Single	Yes	Not stated	Anatomical structures were divided into seven categories: brain, spine, lung, heart, skeletal, gastrointestinal, and genitourinary.<br><br>Overall and individual body organ accuracy rates given.
**Votino, 2018**	GE Voluson E8	6-18MHz linear,<br>6-12MHz curved,<br>5-9MHz curved	15	Fetuses either fixed in formalin (>15 weeks gestation), otherwise no additional preparation.	Fetal medicine doctor, 10 years of ultrasound experience	Single	No	Median time 2 days (1 hour - 4 days)	Diagnoses categorised into three body systems: neurological (brain/spine), thorax (including heart) and abdomen.<br><br>Individual body system accuracy rates given.
**Kang, 2019**	GE Voluson E8;<br><br>GE LOGIQ E9; Samsung HM70A	GE Voluson:<br>6-18MHz linear<br>6-12MHz curved<br>5-9MHz curved<br><br>GE Logiq:<br>2.5 – 8MHz linear	Not stated	No additional preparation over cold storage of body	Fetal medicine doctor or paediatric radiologist (>5years experience each)	Two (one from each centre)	Yes	Median time 2 days (0 – 39 days)	19 internal organs assessed and grouped into 4 anatomic regions for analysis: neurological (brain/spine), thorax, heart and abdomen.

		1 – 5MHz curved<br><br>Samsung:<br>7-16 MHz linear							Overall and individual body system accuracy rates given.

Confidential: For Review Only

Table 4: Details of the reference test for studies included for systematic review.

Author, year	Reference Test	Autopsy Standards	Person(s) performing reference test, experience	Blinded to antenatal clinical history	Blinded to index (ultrasound) test results	Median time between imaging or delivery and autopsy
**Prodhomme, 2015**	Conventional autopsy	Not stated	Pathologists, experience not quantified	No	Not stated	Not stated
**Tuchtan, 2018**	Conventional autopsy	Societé française de foetopathologie (SOFFoeT, France) guidelines	Pathologists, experience not quantified	No	Yes	Not stated
**Votino, 2018**	Conventional autopsy	Societé française de foetopathologie (SOFFoeT, France) guidelines	Pathologists with 20 years' experience	No	Yes	1 day from US to autopsy (1 hour – 2 days)
**Kang, 2019**	Conventional autopsy or minimally invasive autopsy	Conventional autopsy guidelines (SOFFoeT and Royal College of Pathologists)<br>Or<br>Minimally invasive endoscopic methods described by Sebire N et al, 2012	Paediatric pathologists, >15 years' experience	No	Yes	5 days from delivery to autopsy (0 – 47 days)

Confidential: For Review Only

A variety of ultrasound machine vendors and models were used. The most popular ultrasound machine was Voluson E8 (GE Medical Systems, Zipf, Austria), used by 3/5 (60%) centres. High frequency linear probes were used by all operators, with 3/5 (60%) centres also using curvilinear or micro-convex ultrasound probes to acquire two-dimensional (2D) images. One study also acquired three dimensional (3D) ultrasound volumes for head and body imaging in addition to standard 2D views [20].

A single operator performed all post-mortem ultrasound studies in almost all centres, except for one centre (based in Belgium) for the study by Kang et al[17], where multiple operators performed the ultrasound scanning. For all cases the ultrasound was performed prior to autopsy (therefore without knowledge of the reference test results). In two studies (2/4, 50%) the operators were blinded to the antenatal history [17, 19], and in the remainder the antenatal results were known to the operator [18, 20]. The operator was frequently a paediatric radiologist (3/5, 60%) or fetal medicine specialist with ultrasound experience (2/5, 40%).

Reference Test

For all studies the reference standard was conventional autopsy. Only one study described a subset of patients (11/123, 9%) who underwent a minimally invasive autopsy by endoscopic examination [17], using a technique described by Sebire et al [21]. All of the studies reported that their autopsies were performed according to national guidelines (i.e. Royal College of Pathologists [22, 23], SOFFoeT [24], French National Authority of Health [25]). In one study the autopsy guideline was not mentioned [18].

All autopsies were performed by one of several pathologists within a department, rather than a single individual. The pathologists were aware of the clinical and antenatal history for all cases, and in three of the four studies the pathologists were blinded to the ultrasound results.

Methodological Quality Assessment

Using the QUADAS-2 tool, there was a moderate to high risk of bias concerning the 'index text' domain because the persons performing the ultrasound examination in two studies were aware of the patient's antenatal history, and therefore could have been biased when performing the examination [18, 20]. The risk of bias regarding

Confidential: For Review Only

the ‘flow and timing’ domain were unclear for two other studies since timing between ultrasound examination and autopsy were not defined [18, 19].

In one study [18], the risk of bias was unclear for both the patient selection and also reference standard, given that it was not stated how patients were recruited into the study or whether the autopsy results could have been biased by the ultrasound findings. Assessment of bias regarding overall applicability concerns was low **(Figure 2).**

Diagnostic Accuracy of ultrasound by body system

The diagnostic accuracy rates for sensitivity and specificity of each body system are summarised in **Table 5 and Table 6**, with the results given in summarised receiver operator (SROC) plots in **Figures 3 and 4** for overall body diagnoses, and per body system respectively.

Confidential: For Review Only

Table 5: Estimated sensitivities and specificities of different body parts/ systems from studies included in systematic review, with 95% confidence intervals (CI) in square brackets. ND – non-diagnostic, TP – true positive, TN – true negative, FP - false positive, FN – false negative, NA – not applicable

Author, year	Body Part	Total sample size	Sample size with autopsy	Excluded			TP	FN	FP	TN	Sensitivity, % [95% CI] *	Specificity, % [95% CI] *
				ND autopsy	ND PMUS	No autopsy						
**Neurological**												
**Votino, 2018**	Brain	88	66	17	4	5	10	1	7	48	87.5 [59.8, 97.1]	86.6 [75.3, 93.2]
**Prodhomme, 2015**	Brain	169	169	0	0	NA	84	10	0	75	88.9 [81.1, 93.8]	99.3 [94.0, 99.9]
**Tuchtan, 2018**	Brain	75	75	0	0	NA	22	5	1	47	80.4 [62.4, 91.0]	96.9 [87.8, 99.3]
**Prodhomme, 2015**	Spine	169	169	0	0	NA	18	0	0	151	97.4 [79.1, 99.7]	99.6 [96.9, 100.0]
**Tuchtan, 2018**	Spine	75	75	0	0	NA	10	0	0	65	95.5 [67.9, 99.5]	99.2 [93.1, 99.9]
**Kang, 2019**	Brain & Spine	123	70	53	13	NA	13	8	11	38	61.4 [40.8, 78.5]	77.0 [63.7, 86.5]
**Cardiothoracic**												
**Prodhomme, 2015**	Thoracic	169	169	0	0	NA	10	1	0	158	87.5 [59.8, 97.1]	99.7 [97.1, 100.0]
**Kang, 2019**	Thoracic	123	123	0	21	NA	5	14	20	84	27.5 [12.8, 49.4]	80.4 [71.9, 87.0]
**Tuchtan, 2018**	Thoracic	75	75	0	0	NA	6	2	0	67	72.2 [40.2, 91.0]	99.3 [93.3, 99.9]
**Prodhomme, 2015**	Cardiac	169	169	0	0	NA	2	9	0	158	20.8 [6.7, 49.1]	99.7 [97.1, 100.0]
**Kang, 2019**	Cardiac	123	122	1	24	NA	13	13	18	78	50.0 [32.4, 67.6]	81.0 [72.0, 87.5]
**Tuchtan, 2018**	Cardiac	75	75	0	0	NA	4	18	0	53	19.6 [8.3, 39.5]	99.1 [91.7, 99.9]
**Votino, 2018**	Cardiac & Thoracic	88	88	0	2	NA	16	2	5	65	86.8 [65.5, 95.8]	92.3 [83.7, 96.5]

Confidential: For Review Only

**Abdominal**												
**Votino, 2018**	Whole Abdomen	88	88	0	2	NA	12	2	4	70	83.3 [58.4, 94.7]	94.0 [86.2, 97.5]
**Kang, 2019**	Whole Abdomen	123	123	0	19	NA	17	11	23	72	60.3 [42.4, 75.9]	75.5 [66.0, 83.0]
**Prodhomme, 2015**	Gastro-intestinal	169	169	0	0	NA	15	10	0	144	59.6 [40.7, 76.0]	99.7 [96.8, 100.0]
**Tuchtan, 2018**	Gastro-intestinal	75	75	0	0	NA	2	0	1	72	83.3 [31.0, 98.2]	98.0 [91.7, 99.5]
**Prodhomme, 2015**	Urinary System	169	169	0	0	NA	37	2	0	130	93.7 [81.8, 98.0]	99.6 [96.4, 100.0]
**Tuchtan, 2018**	Urinary System	75	75	0	0	NA	9	1	0	65	86.4 [57.1, 96.8]	99.2 [93.1, 99.9]

*Sensitivities, specificities and associated 95% confidence intervals are calculated using the standard univariate approach while making an adjustment of 0.5 for zero frequencies where necessary.

Confidential: For Review Only

Table 6: Estimates of mean sensitivity and specificity (with 95% confidence intervals), overall and split by different body systems

**Body System**	**Mean sensitivity [95% CI]**	**Mean specificity [95% CI]**
Overall	73.3 [59.9, 83.5]	96.6 [92.6, 98.4]
Neurological	84.3 [70.8, 92.2]	96.7 [86.5, 99.3]
Cardiothoracic	52.1 [27.6, 75.5]	96.6 [86.8, 99.2]
Abdominal	78.4 [61.0, 89.4]	97.3 [88.9, 99.4]
p-value (neuro vs. cardio)	0.010	0.916
p-value (neuro vs. abdom)	0.498	0.819
p-value (cardio vs. abdom)	0.059	0.731

Confidential: For Review Only

Two studies did not explicitly state the non-diagnostic imaging or non-diagnostic autopsies rate [18, 19]. Kang et al [17] reported a non-diagnostic ultrasound result for 13/70 (18.6%) brain, 21/123 (17.1%) thoracic, 24/122 (19.7%) cardiac and 19/123 (15.4%) abdominal examinations. The non-diagnostic autopsy rate was 53/123 (43.1%) for the brain and 1/123 (0.8%) for the heart.

Votino et al[20] reported non-diagnostic ultrasound imaging in 4/62 (6.5%) brain and 2/86 (2.3%) cardiothoracic ultrasound examinations. The non-diagnostic autopsy rate was 17/88 (19.3%) for the brain. In 5/88 (5.7%) cases, the brain was not examined at autopsy, only the body.

Four studies were included in the bivariate meta-analysis model, all related to ultrasound usage in perinatal deaths. There was little evidence that studies had different thresholds for diagnosis (i.e. negative correlation between sensitivity and specificity) (Spearman's correlation (rho) = -0.310; 95% CI -0.670, 0.168). The mean diagnostic sensitivity and specificity respectively (reported in table 6) was 84.3% (95% CI: 70.8%, 92.2%) and 96.7% (95% CI: 86.5%, 99.3%) for neurological abnormalities; 52.1% (95% CI: 27.6%, 75.5%,) and 96.6% (95% CI: 86.8%, 99.2%) for cardiothoracic abnormalities and 78.4% (95% CI: 61.0%, 89.4%) and 97.3% (95% CI: 88.9%, 99.4%) for abdominal abnormalities. Combining all body systems, the mean sensitivity was 73.3% (95% CI: 59.9%, 83.5%) and the mean specificity was 96.6% (95% CI: 92.6%, 98.4%). Mean diagnostic sensitivity in the cardiothoracic body system was significantly lower than the neurological system (p=0.010) and marginally lower than the abdominal system, although non-significant at the 5% level (p-0.059).

The study by Kang et al [14] appeared to be an outlier compared to the other three studies, particularly for specificity rates of all body systems. When excluding this outlier the mean diagnostic sensitivity and specificity respectively was 87.4% (95% CI: 79.5%, 92.5%) and 97.9% (95% CI: 90.7%, 99.6%) for neurological abnormalities; 57.8% (95% CI: 24.1%, 85.6%) and 98.7% (95% CI: 94.5, 99.7) for cardiothoracic abnormalities and 82.3% (95% CI: 63.8%, 92.5%) and 98.4% (95% CI: 94.5%, 99.5%) for abdominal abnormalities. Combining all body systems, the mean sensitivity was 78.8% (95% CI: 64.6%, 88.4%) and the mean specificity was 98.3% (95% CI: 96.3%, 99.2%). See **Appendix, Figures S2 and S3** for the SROC curves for overall diagnoses and by body system, with this study excluded. .

Confidential: For Review Only

DISCUSSION:

Post-mortem ultrasound for perinatal deaths is a feasible technique, and currently performed in a few European centres. The diagnostic accuracy rates are highest for neurological and abdominal abnormalities (mean sensitivity rates of 84.3% and 78.4% respectively), but less effective for cardiothoracic abnormalities (mean sensitivity rate of 52.1%). There were no studies reporting the post-mortem ultrasound accuracy rates in neonates and older children.

The study raises two main potential clinical implications, firstly relating to improved parental choice and secondly potential healthcare cost savings. It is well known that parents report a high acceptability for non-invasive autopsy methods [3, 26, 27], and that many healthcare professionals also advocate post-mortem imaging techniques [28]. Having another imaging modality available, such as ultrasound, would likely increase options for parental choice and their access to imaging. It may serve as a means for opening channels of communication between parents and clinicians, possibly reduce time spent grieving by offering a diagnosis and form of closure, or highlight the need for further counselling. As pregnant mothers will ubiquitously have been subject to fetal or antenatal ultrasound, they may have better inherent understanding of post-mortem ultrasound rather than cross-sectional imaging techniques.

In terms of finances, a previous systematic review by Ahmad et al [4] described a potential cost saving with MRI virtopsy compared to invasive autopsy of approximately 33% (based on a full body MRI costing £226.34 and an autopsy costing £471.80, with a paediatric MRI virtopsy sensitivity rate of 73%). Using their same reference for costing [29], an ultrasound study taking approximately 20 minutes (costing £56 per case, with similar whole body ultrasound pooled sensitivity rate of 74%), could offer even further benefits. Nevertheless, this calculation is based on assuming a similar rate of referral for invasive autopsy, which may be unlikely given the potential higher parental acceptability. Whether the balance between increased referrals and reduction in cost per patient would negate potential savings remains to be seen.

The comparatively low post-mortem ultrasound sensitivity rates for cardiothoracic abnormalities is also important to recognise, and may warrant alternative imaging with higher sensitivity (e,g, PMMR; 60% - 82% [30, 31]). Whether detection of abnormalities with ultrasound will improve with operator experience, or is related to the technique itself is unknown. The lack of circulation and inability to utilise Doppler imaging on ultrasound,

Confidential: For Review Only

teamed with the presence of post mortem intracardiac gas may hamper sonographic visualisation of cardiac structural abnormalities but 3D post-mortem ultrasound may possibly resolve some of these issues in future studies. In addition, PMUS operators may undergo a learning curve similar to that found for thoracic abnormalities using PMMR, with improvements in diagnostic accuracy from 30 – 37% in early reports [32] to 81.8%[31] later. This may relate to better knowledge of normal post-mortem changes on imaging (i.e. not overcalling pleural effusions as pathological) although infective pathologies still remain difficult to detect.

In this systematic review, the majority of studies reported similar ultrasound diagnostic accuracy rates although those by Kang et al [17] were much lower, particularly for sensitivity rates of neurological and thoracic abnormalities and, specificity rates for all body systems. Reasons for this may relate to the fact that non-diagnostic ultrasound results were considered false negative for sensitivity calculation, and false positives for specificity calculations. In addition, the authors did report a high non-diagnostic ultrasound and autopsy rate for brain imaging of approximately 13/70 (18.6%) and for thoracic imaging of 21/123 (17.1%). While demographic details of the perinatal deaths in their study were similar to others, the high non-diagnostic rate may suggest a greater referral of macerated fetuses, which could have impeded ultrasound visualisation of certain organs and thus accurate diagnosis. There was also a laparoscopic assisted minimally invasive autopsy as the reference standard in a subset of cases (11/123, 9%) [21]. Whilst this technique has been shown to provide a high success rate in terms of tissue sampling and detection of histological abnormalities, the true diagnostic accuracy rate compared to conventional autopsy remains unknown [33]. Despite these factors, we still include the results in our meta-analysis given that this was the only multicentre trial in our review and described results from more than one ultrasound operator, thereby providing potentially a more generalisable result for post-mortem ultrasound outcomes.

There were several limitations to this study. The first relates to the generalizability of our results. There were very few studies assessing the diagnostic accuracy of post-mortem ultrasound in children, with all studies focussing on perinatal deaths, conducted within Europe, and with ultrasound examinations being performed by the same operator within each centre. The examinations were conducted within a few specialist centres focussing on this niche area, meaning they may represent an elevated level of accuracy, potentially not achievable if post-mortem ultrasound was to be offered in a more general clinical setting.

Confidential: For Review Only

The second limitation includes the classification and detection of abnormalities. In all cases the pathologists performing the autopsies were not blinded to the antenatal history, and in one of the studies the ultrasound operator was also unblinded. There may have been a variation in the way post-mortem ultrasounds are performed and reported. For example, some operators may have considered ascites and pleural effusions as part of normal post-mortem change [34, 35], whilst others may view these as significant abnormalities. There may also have been variation regarding the recording of non-diagnostic ultrasound examinations. In 2 studies the number of non-diagnostic autopsies and ultrasound examinations were not reported [18, 19], and it is not clear whether those ultrasound examinations were all of diagnostic quality, whether these were excluded prior to analysis, or whether the authors simply recorded these as 'no abnormality' where no significant findings were found, regardless of diagnostic quality. In the other two papers where non-diagnostic ultrasound examinations were reported, one excluded these from analysis [17] whereas the other considered them as an inaccurate finding (i.e. recorded as 'false positive')[20]. Nevertheless despite such limitations, our work has several strengths. This paper represents the first systematic review on use of post-mortem ultrasound in perinatal deaths. It demonstrates a reasonable diagnostic accuracy for the majority of body systems and that it can be performed on a variety of scanner models, in a varied cohort of perinatal deaths across a range of gestational ages.

Currently there are no standardized protocols on how best to perform a post-mortem ultrasound study, nor for which situations in would be most beneficial in helping to change clinical management for future pregnancies. For example, it may have an apparent higher diagnostic rate in fetal demise for those pregnancies who have not yet undergone detailed second or third trimester antenatal ultrasound (i.e after 20 weeks gestation).

Further research should be direct towards PMUS studies involving a larger cohort of perinatal deaths and also assessing usage in older children. There is clearly potential for this technique to be adopted in different clinical settings (such as in the developing world where pathological expertise is lacking but where causes of death are still important to confirm to inform global public health initiatives [36]), by other specialists to increase appeal and availability following adequate training (e.g. pathologists, obstetricians, sonographers) and international guidelines should be established for the appropriate indications for usage. There is also the ability for ultrasound to be used as a tool to aid percutaneous organ biopsies where tissue sampling is required to confirm histological diagnoses. In conclusion, this systematic review has provided evidence in showing that it is a feasible technique

Confidential: For Review Only

with a reasonable diagnostic accuracy when performed by experienced operators, however further work will enable a better understanding of its place and purpose within a future non-invasive autopsy service.

ACKNOWLEDGEMENTS:

1. **Conflict of Interest:**

   All authors have completed the ICMJE uniform disclosure forms and declare: no support from any organisation for the submitted work; no financial relationships with any organisations that might have an interest in the submitted work in the previous three years; no other relationships or activities that could appear to have influenced the submitted work.

2. **Role of Funding Source**

   The funder of the study had no role in the study design, data collection, data analysis, data interpretation, or writing of the report. The corresponding author and chief investigator had full access to all the data in the study and final responsibility for decision to submit for publication.

3. **Ethical Approval:**

   Institutional review board approval was not required because it comprises a systematic review of published literature.

4. **Informed Consent:**

   Written informed consent was not required for this study because it comprises a systematic review of published literature.

5. **Funding**

   SCS is supported by a RCUK/ UKRI Innovation Fellowship and Medical Research Council (MRC) Clinical Research Training Fellowship (Grant Ref: MR/R00218/1). This award is jointly funded by the Royal College of Radiologists (RCR). OJA is funded by a National Institute for Health Research (NIHR) Career Development Fellowship (NIHR-CDF-2017-10-037), and NJS funded by an NIHR Senior Investigator award.

Confidential: For Review Only

The authors receive funding from the Great Ormond Street Children's Charity and the Great Ormond Street Hospital NIHR Biomedical Research Centre. This article presents independent research funded by the MRC, RCR, NIHR and the views expressed are those of the author(s) and not necessarily those of the NHS, MRC, RCR, the NIHR or the Department of Health.

6. Author Contribution

All authors listed in this manuscript fulfil the ICMJE recommendations for authorship. All authors provided substantial contribution to the conception and design of the work, analysis and interpretation; and drafting the work for intellectual content. All authors have had final approval for the version to be published and agree to be accountable for all aspects of the work in ensuring that questions related to the accuracy are appropriately investigated and resolved.

7. Data Sharing Statement

All data is provided in full in the results section of this paper and in the appendices for search terminologies. Raw numbers provided in an excel spreadsheet are available upon request from the main author.

Confidential: For Review Only

FIGURE LEGENDS:

Figure 1

PRISMA flow chart for the study search and selection

Figure 2

Methodological quality assessment of the included studies using the QUADAS-2 tool. Risk of bias and applicability concerns summary about each domain are shown for each included study

Figure 3

ROC plot of sensitivity against false positivity rate (1 – specificity) of all 19 studies from table 5. Bivariate overall summary estimates of sensitivity and false positivity rate are overlaid with corresponding 95% confidence ellipses.

Figure 4

ROC plot of sensitivity against false positivity rate (1 – specificity) of 19 studies from table 5 separated by body system. Bivariate overall summary estimates of sensitivity and false positivity rate for each body system are overlaid with corresponding 95% confidence ellipses.

Confidential: For Review Only

REFERENCES:

1. Sieswerda-Hoogendoorn T, van Rijn RR. Current techniques in postmortem imaging with specific attention to paediatric applications. *Pediatr Radiol* 2010; **40**: 141-152; quiz 259. DOI: 10.1007/s00247-009-1486-0.

2. Arthurs OJ, van Rijn RR, Sebire NJ. Current status of paediatric post-mortem imaging: an ESPR questionnaire-based survey. *Pediatr Radiol* 2014; **44**: 244-251. DOI: 10.1007/s00247-013-2827-6.

3. Lewis C, Hill M, Arthurs OJ, Hutchinson C, Chitty LS, Sebire NJ. Factors affecting uptake of postmortem examination in the prenatal, perinatal and paediatric setting. *Bjog* 2018; **125**: 172-181. DOI: 10.1002/uog.17468 10.1111/1471-0528.14600.

4. Ahmad MU, Sharif KA, Qayyum H, Ehsanullah B, Balyasnikova S, Wale A, Shanmuganandan A, Siddiqui MRS, Athanasiou T, Kemp GJ. Assessing the use of magnetic resonance imaging virtopsy as an alternative to autopsy: a systematic review and meta-analysis. *Postgrad Med J* 2017; **93**: 671-678. DOI: 10.1136/postgradmedj-2017-134945.

5. van Rijn RR, Beek EJ, van de Putte EM, Teeuw AH, Nikkels PGJ, Duijst W, Nievelstein RA, Dutch NG. The value of postmortem computed tomography in paediatric natural cause of death: a Dutch observational study. *Pediatr Radiol* 2017; **47**: 1514-1522. DOI: 10.1007/s00247-017-3911-0.

6. Arthurs OJ, Guy A, Thayyil S, Wade A, Jones R, Norman W, Scott R, Robertson NJ, Jacques TS, Chong WK, Gunny R, Saunders D, Olsen OE, Owens CM, Offiah AC, Chitty LS, Taylor AM, Sebire NJ. Comparison of diagnostic performance for perinatal and paediatric post-mortem imaging: CT versus MRI. *Eur Radiol* 2016; **26**: 2327-2336. DOI: 10.1007/s00330-015-4057-9.

7. Oyake Y, Aoki T, Shiotani S, Kohno M, Ohashi N, Akutsu H, Yamazaki K. Postmortem computed tomography for detecting causes of sudden death in infants and children: retrospective review of cases. *Radiat Med* 2006; **24**: 493-502. DOI: 10.1007/s11604-006-0061-y.

8. Sieswerda-Hoogendoorn T, Soerdjbalie-Maikoe V, de Bakker H, van Rijn RR. Postmortem CT compared to autopsy in children; concordance in a forensic setting. *Int J Legal Med* 2014; **128**: 957-965. DOI: 10.1007/s00414-014-0964-6.

9. Shelmerdine SC, Langan D, Hutchinson JC, Hickson M, Pawley K, Suich J, Palm L, Sebire NJ, Wade A, Arthurs OJ. Chest radiographs versus CT for the detection of rib fractures in children (DRIFT): a diagnostic accuracy observational study. *The Lancet Child & adolescent health* 2018; **2**: 802-811. DOI: 10.1016/s2352-4642(18)30274-8.

10. Prodhomme O, Baud C, Saguintaah M, Béchard-Sevette N, Bolivar J, David S, Taleb-Arrada I, Couture A. Principles of fetal postmortem ultrasound: A personal review. *Journal of Forensic Radiology and Imaging* 2015; **3**: 12-15. DOI: 10.1016/j.jofri.2015.01.008.

11. Kumar M, Priyam. Ultrasonography and autopsy correlation of fetal hypoplastic left heart syndrome. *Journal of clinical ultrasound : JCU* 2018. DOI: 10.1002/jcu.22607.

12. Shelmerdine SC. Diagnostic accuracy of paediatric post-mortem ultrasound compared with autopsy: a systematic review. https://www.crd.york.ac.uk/prospero/display_record.php?RecordID=106968 [1 Sept 2018]

13. Moher D, Liberati A, Tetzlaff J, Altman DG, Group P. Preferred reporting items for systematic reviews and meta-analyses: the PRISMA statement. *BMJ (Clinical research ed)* 2009; **339**: b2535. DOI: 10.1136/bmj.b2535.

14. Whiting PF, Rutjes AW, Westwood ME, Mallett S, Deeks JJ, Reitsma JB, Leeflang MM, Sterne JA, Bossuyt PM. QUADAS-2: a revised tool for the quality assessment of diagnostic accuracy studies. *Annals of internal medicine* 2011; **155**: 529-536. DOI: 10.7326/0003-4819-155-8-201110180-00009.

Confidential: For Review Only

15. Team RC. R: A language and environment for statistical computing. R Foundation for Statistical Computing: Vienna, Austria, 2016.

16. Doebler P. Meta-Analysis of Diagnostic Accuracy with mada. R package version 0.5.9. www.r-forge.r-project.org/projects/mada/

17. Kang X, Shelmerdine SC, Hurtado I, Bevilacqua E, Hutchinson C, Mandalia U, Segers V, Cos Sanchez T, Cannie MM, Carlin A, Sebire NJ, Arthurs OJ, Jani JC. Postmortem examination of human fetuses: a comparison of 2-dimensional ultrasound with invasive autopsy. *Ultrasound Obstet Gynecol* 2019. Feb; 53(2):229-238 DOI: 10.1002/uog.18828.

18. Prodhomme O, Baud C, Saguintaah M, Béchard-Sevette N, Bolivar J, David S, Taleb-Arrada I, Couture A. Comparison of postmortem ultrasound and X-Ray with autopsy in fetal death: Retrospective study of 169 cases. *Journal of Forensic Radiology and Imaging* 2015; **3**: 120-130. DOI: 10.1016/j.jofri.2015.04.002.

19. Tuchtan L, Lesieur E, Bartoli C, Delteil C, Sarda-Quarello L, Torrents J, Sigaudy S, Piercecchi MD, Gorincour G. Diagnosis of congenital abnormalities with post-mortem ultrasound in perinatal death. *Diagnostic and interventional imaging* 2018; **99**: 143-149. DOI: 10.1016/j.diii.2017.11.005.

20. Votino C, Cos Sanchez T, Bessieres B, Segers V, Kadhim H, Razavi F, Condorelli M, Votino R, D'Ambrosio V, Jani J. Minimally invasive fetal autopsy using ultrasound: a feasibility study. *Ultrasound in Obstetrics and Gynecology* 2018; **52**: 776-783.

21. Sebire NJ, Weber MA, Thayyil S, Mushtaq I, Taylor A, Chitty LS. Minimally invasive perinatal autopsies using magnetic resonance imaging and endoscopic postmortem examination ("keyhole autopsy"): feasibility and initial experience. *J Matern Fetal Neonatal Med* 2012; **25**: 513-518. DOI: 10.3109/14767058.2011.601368.

22. Royal College of Pathologists. Guidelines on autopsy practice: Fetal autopsy (2nd trimester fetal loss and termination of pregnancy for congenital anomaly). www.rcpath.org [Accessed 16 May 2018].

23. Royal College of Pathologists. Guidelines on autopsy practice: Third trimester antepartum and intrapartum stillbirth. www.rcpath.org [Accessed 16 May 2018].

24. Societe Francaise de Foetopathologie. http://soffoet.fr/page-d-exemple/la-foetopathologie/ [Accessed 09 January 2019].

25. Haute Autorite de Sante. https://www.has-sante.fr/portail/jcms/r_1455081/fr/home-page?portal=r_1455081 [Accessed 9th January 2019].

26. Kang X, Cos T, Guizani M, Cannie MM, Segers V, Jani JC. Parental acceptance of minimally invasive fetal and neonatal autopsy compared with conventional autopsy. *Prenat Diagn* 2014; **34**: 1106-1110. DOI: 10.1002/pd.4435.

27. Cannie M, Votino C, Moerman P, Vanheste R, Segers V, Van Berkel K, Hanssens M, Kang X, Cos T, Kir M, Balepa L, Divano L, Foulon W, De Mey J, Jani J. Acceptance, reliability and confidence of diagnosis of fetal and neonatal virtuopsy compared with conventional autopsy: a prospective study. *Ultrasound Obstet Gynecol* 2012; **39**: 659-665. DOI: 10.1002/uog.10079.

28. Lewis C, Hill M, Arthurs OJ, Hutchinson JC, Chitty LS, Sebire N. Health professionals' and coroners' views on less invasive perinatal and paediatric autopsy: a qualitative study. *Archives of disease in childhood* 2018. DOI: 10.1136/archdischild-2017-314424.

29. NHS National Tariff Payment System 2016/17. Annex A. https://www.gov.uk/government/publications/nhs-national-tariff-payment-system-201617 [Accessed 24 January 2019].

30. Taylor AM, Sebire NJ, Ashworth MT, Schievano S, Scott RJ, Wade A, Chitty LS, Robertson N, Thayyil S. Postmortem cardiovascular magnetic resonance imaging in fetuses and children: a masked

Confidential: For Review Only

comparison study with conventional autopsy. *Circulation* 2014; **129**: 1937-1944. DOI: 10.1161/circulationaha.113.005641.

31. Ashwin C, Hutchinson JC, Kang X, Langan D, Jones R, Norman W, Cannie M, Jani J, Sebire NJ, Arthurs OJ. Learning effect on perinatal post-mortem magnetic resonance imaging reporting: single reporter diagnostic accuracy of 200 cases. *Prenat Diagn* 2017; **37**: 566-574. DOI: 10.1002/pd.5043.

32. Arthurs OJ, Thayyil S, Olsen OE, Addison S, Wade A, Jones R, Norman W, Scott RJ, Robertson NJ, Taylor AM, Chitty LS, Sebire NJ, Owens CM. Diagnostic accuracy of post-mortem MRI for thoracic abnormalities in fetuses and children. *Eur Radiol* 2014; **24**: 2876-2884. DOI: 10.1007/s00330-014-3313-8.

33. Hutchinson C, Shelmerdine SC. Feasibility and experience of the MinImAL procedure: Minimally Invasive perinatal and paediatric Autopsies with Laparoscopically assisted tissue sampling. 2019. DOI: 10.1002/uog.20211.

34. Arthurs OJ, Barber JL, Taylor AM, Sebire NJ. Normal perinatal and paediatric postmortem magnetic resonance imaging appearances. *Pediatr Radiol* 2015; **45**: 527-535. DOI: 10.1007/s00247-014-3166-y.

35. Barber JL, Hutchinson JC, Sebire NJ, Arthurs OJ. Pleural fluid accumulation detectable on paediatric post-mortem imaging: a possible marker of interval since death? 2016; **130**: 1003-1010. DOI: 10.1007/s00414-016-1320-9.

36. Menendez C, Castillo P, Martinez MJ, Jordao D, Lovane L, Ismail MR, Carrilho C, Lorenzoni C, Fernandes F, Nhampossa T, Hurtado JC, Navarro M, Casas I, Santos Ritchie P, Bandeira S, Mocumbi S, Jaze Z, Mabota F, Munguambe K, Maixenchs M, Sanz A, Mandomando I, Nadal A, Gonce A, Munoz-Almagro C, Quinto L, Vila J, Macete E, Alonso P, Ordi J, Bassat Q. Validity of a minimally invasive autopsy for cause of death determination in stillborn babies and neonates in Mozambique: An observational study. *PLoS Med* 2017; **14**: e1002318. DOI: 10.1371/journal.pmed.1002318.

Confidential: For Review Only

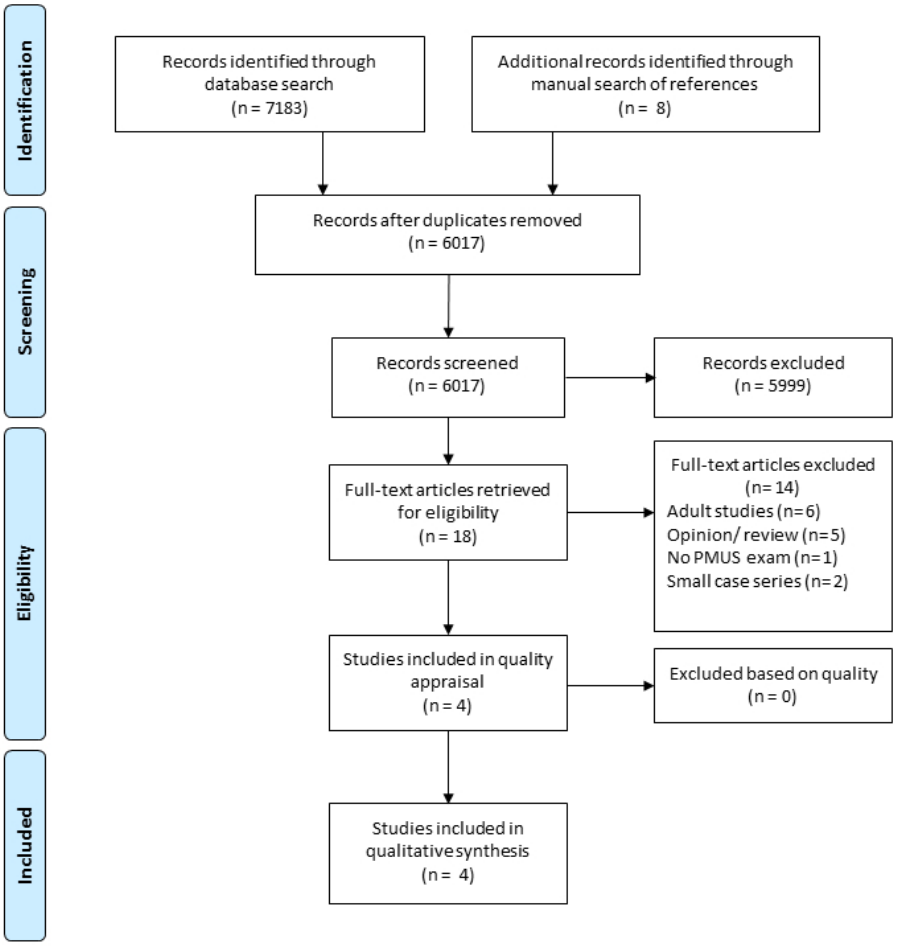

200x190mm (96 x 96 DPI)

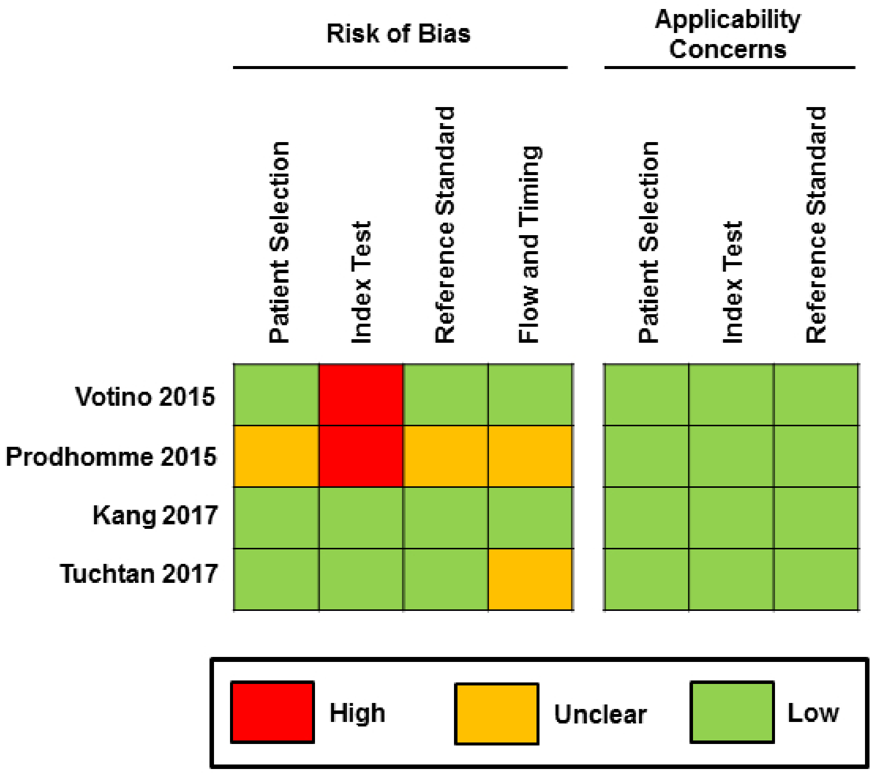

167x149mm (96 x 96 DPI)

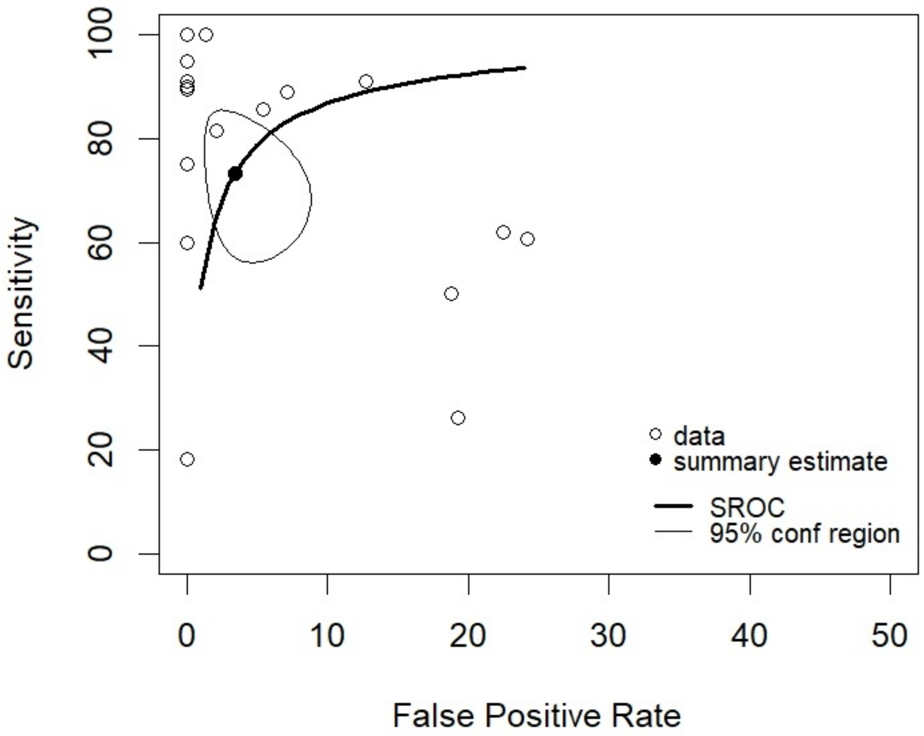

127x101mm (150 x 150 DPI)

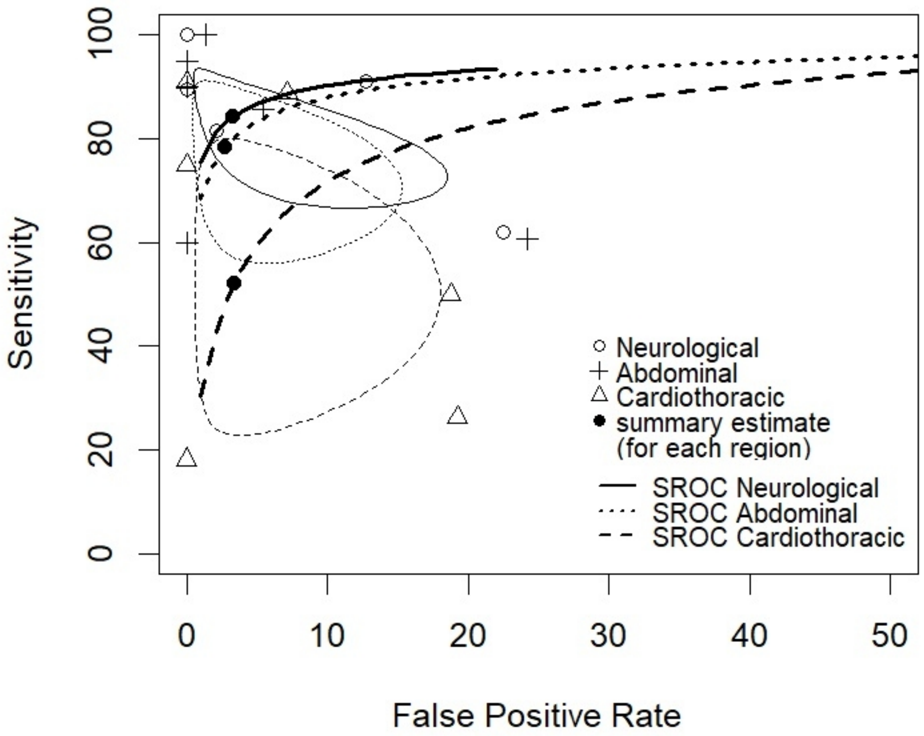

127x101mm (150 x 150 DPI)

Appendix

S1. Search strategy for retrieval of included studies

MEDLINE:

#	01 January 1998 to 31 December 2018	Results (n)
1	(Autopsy [Mesh] OR autopsy [tiab] OR necropsy [tiab] OR post-mortem [tiab] OR postmortem [tiab])	148316
2	(Infant death, sudden [Mesh] OR infant* [tiab] OR child*[tiab] OR adolescent [tiab] OR pediatric*[tiab] OR paediatric*[tiab] OR neonate*[tiab] OR newborn*[tiab] OR baby [tiab] OR babies [tiab] OR youth*[tiab] OR perinatal OR fetal [tiab] OR foetal [tiab] OR termination* [tiab] OR abortion* [tiab])	2276140
3	("Ultrasonography"[Mesh] OR ultraso*[tiab] OR sonograph*[tiab] OR echo*[tiab] OR imaging [tiab] OR radiology [tiab])	1308153
**4**	1 and 2 and 3	**3133**

EMBASE:

#	01 January 1998 to 31 December 2018; Human subjects only	Results (n)
1	Autopsy.a.f	68338
2	(postmortem or post-mortem).a.f	33839
3	1 or 2	87723
4	(child* or preschool child* or adolescent or infant* or newborn* or pediatric* or infant* or newborn* or neonat* or baby or babies or paediatric* or adoles* or teen* or youth* or fetal or foetal).a.f.	2900920
5	(ultraso* or sonograph* or echo*).a.f.	778476
**6**	**3 and 4 and 5**	**3915**

COCHRANE LIBRARY

#	01 January 1998 to 31 December 2018	Results (n)
1	Autopsy OR postmortem OR post-mortem (Word variations have been searched)	1181
2	child OR adolescent OR infant OR newborn OR pediatric OR neonate OR baby OR babies OR paediatric OR teen OR youth OR fetal OR foetal (Word variations have been searched)	240101
3	Ultrasonography OR ultraso* OR sonograph* OR echo* OR imaging OR radiology (Word variations have been searched)	102777
**4**	**#1 and #2 and #3**	**135**

Confidential: For Review Only

S2. SROC plot of sensitivity against false positivity rate (1 – specificity) for all studies (excluding the publication by Kang et al 2019) for diagnostic accuracy rate for overall diagnosis. Bivariate overall summary estimates of sensitivity and false positivity rate are overlaid with corresponding 95% confidence ellipses

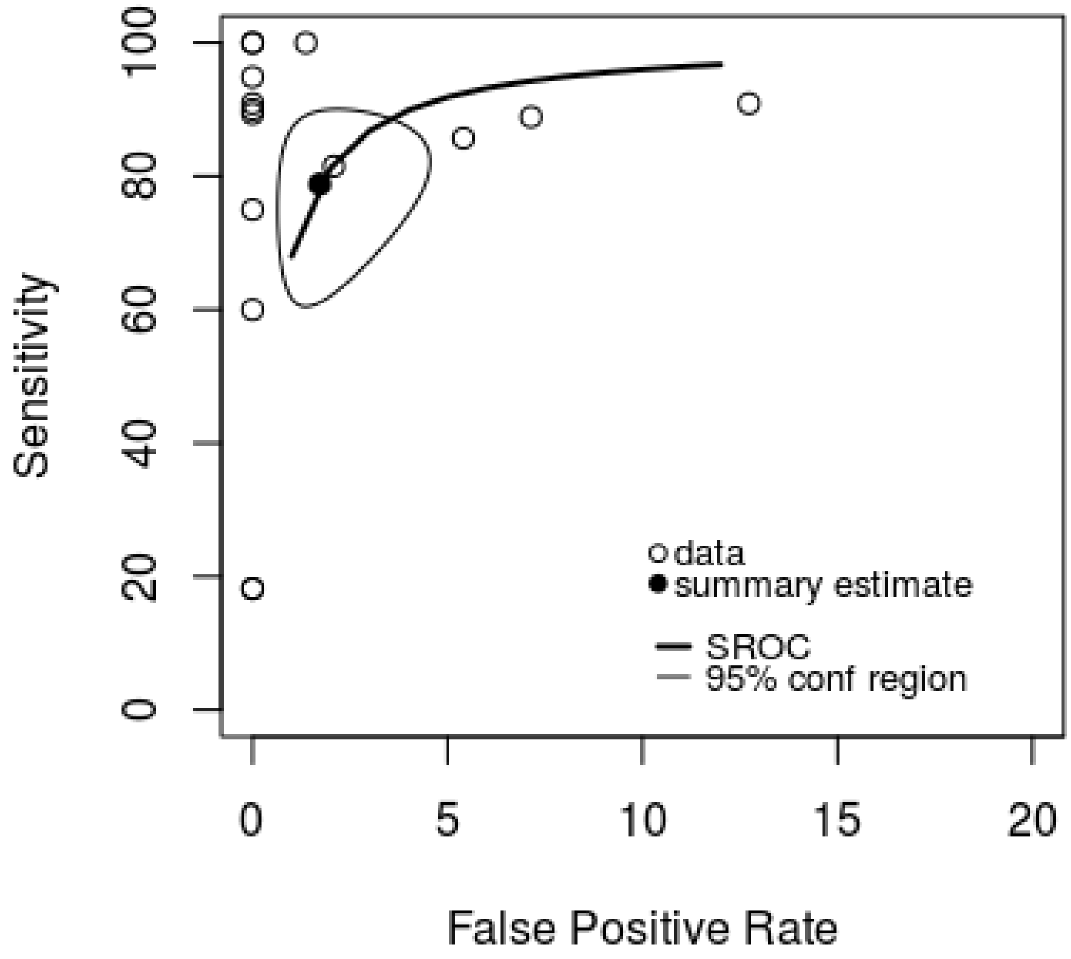

...ew Only

S3. SROC plot of sensitivity against false positivity rate (1 – specificity) for all studies (excluding the publication by Kang et al 2019) for diagnostic accuracy results per body system. Bivariate overall summary estimates of sensitivity and false positivity rate are overlaid with corresponding 95% confidence ellipses.

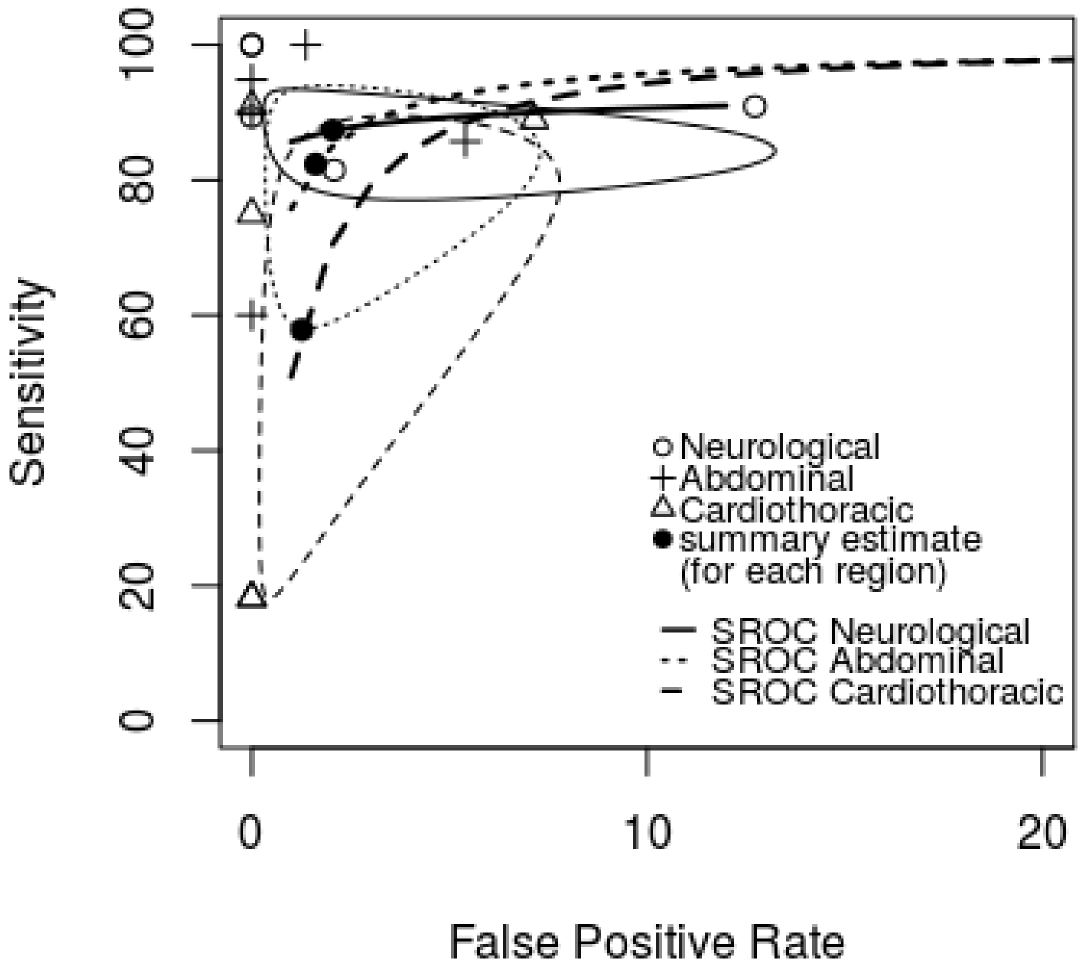

ew Only
